# Spatial–Temporal EEG Imaging for Dual-Loop Neuro-Adaptive Simulation: Cognitive-State Decoding and Communication Gating in Critical Human–Machine Teams

**DOI:** 10.3390/jimaging12050208

**Published:** 2026-05-12

**Authors:** Rubén Juárez, Antonio Hernández-Fernández, Claudia Barros Camargo, David Molero

**Affiliations:** 1 Department of Pedagogía, Faculty of Humanities and Educational Sciences, University of Jaén, 23071 Jaén, Spain; ahernand@ujaen.es; 2 Department of Research Methods and Diagnosis in Education I (MIDE I), Faculty of Education, National University of Distance Education (UNED), 28040 Madrid, Spain; claudia.barros@edu.uned.es; 3 Research Group “Lifelong Education, Neuropedagogical Integration (LE:NI)”, University of Jaén, 23071 Jaén, Spain

**Keywords:** spectral-topographic EEG, cognitive-state decoding, multimodal synchronization, CNN–LSTM, MAPPO, communication gating, haptic guidance, human–machine teaming, cognitive load index, neural privacy

## Abstract

Human performance in critical environments is frequently degraded by mistimed communication delivered during periods of visual–cognitive saturation. In such settings, failures arise not only from individual limitations but also from poor coordination between operators under rapidly changing workload conditions. We present a dual-loop neuro-adaptive simulation framework based on real-time spectral–topographic EEG representations, in which multichannel cortical activity is transformed into dynamic spatial maps and decoded to regulate both operator assistance and team communication. The system integrates 14-channel wireless EEG (Emotiv EPOC X, 256 Hz), gaze tracking, telemetry, and communication events through an LSL-based multimodal synchronization pipeline. A hybrid CNN–LSTM model processes sequences of spectral-topographic EEG maps to classify three operationally actionable neurocognitive states—Channelized Attention, Diverted Attention, and Surprise/Startle—while also estimating a continuous Cognitive Load Index (CLI). These representation-derived features are then used by a multi-agent proximal policy optimization (MAPPO) controller to generate two coordinated outputs: (i) adaptive haptic guidance for the pilot, designed to reduce reliance on overloaded visual and auditory channels, and (ii) a traffic-light communication gate for the telemetry engineer, regulating whether radio intervention should proceed, be delayed, or be withheld. In a high-fidelity dual-station simulation with 25 pilot–engineer pairs, the proposed framework was associated with a reduction of more than 30% in communication breakdown errors relative to open-loop telemetry, with the strongest effects observed during peak-load windows, while preserving realistic task progression. It also improved pilot reaction time to time-critical warnings and reduced engineer decision load under the tested conditions. These findings support the use of spectral-topographic EEG representations as a practical basis for combining multimodal neurophysiological sensing, spatiotemporal pattern decoding, and adaptive coordination in high-pressure human–machine teams. At the same time, the study should be interpreted as evidence of controlled feasibility in a simulated setting rather than as definitive proof of field-level generalization. We further discuss deployment constraints and propose privacy-by-design safeguards to ensure that neurocognitive signals are used exclusively for operational adaptation rather than employability assessment or performance scoring.

## 1. Introduction

Human performance in critical environments increasingly depends on the timely coordination of small teams operating under severe temporal pressure. This challenge is especially evident in pilot–engineer interaction settings, where the operator must maintain continuous control while a remote or co-located support role monitors telemetry, task progression, and emerging hazards. In such settings, failures are often caused not only by task complexity itself but also by the mistimed delivery of information when operators are already under visual–cognitive saturation. From a human-factors perspective, this problem is consistent with split-attention effects and multiple-resource limitations, in which visual control, hazard perception, and auditory instructions compete for bounded working memory and attentional resources [1,2]. Beyond this cognitive interpretation, however, the problem also raises a representation challenge: how to transform rapidly evolving neurophysiological activity into spatially structured patterns that can support real-time operational intervention.

Recent advances in portable neurophysiological sensing have made it possible to acquire electroencephalography (EEG) in ecologically valid settings, opening new opportunities for real-time cognitive-state estimation [3]. Yet, many existing passive brain–computer interface (pBCI) systems remain limited to single-user adaptation and conventional channel-wise feature extraction. By contrast, representing EEG activity as spectral-topographic EEG maps provides a richer basis for modeling cortical dynamics, particularly when the spatial distribution of band-limited neural activity is treated as a time-varying field rather than as a set of isolated electrode measurements. Although such maps should not be conflated with high-density source-resolved neuroimaging, they provide structured spatial representations that can be processed with image-like learning pipelines under realistic wearable-sensing constraints. This perspective emphasizes representation, spatial organization, and temporal evolution, thereby enabling analysis pipelines that go beyond raw signal monitoring toward interpretable spatiotemporal pattern decoding.

**Figure 1:**
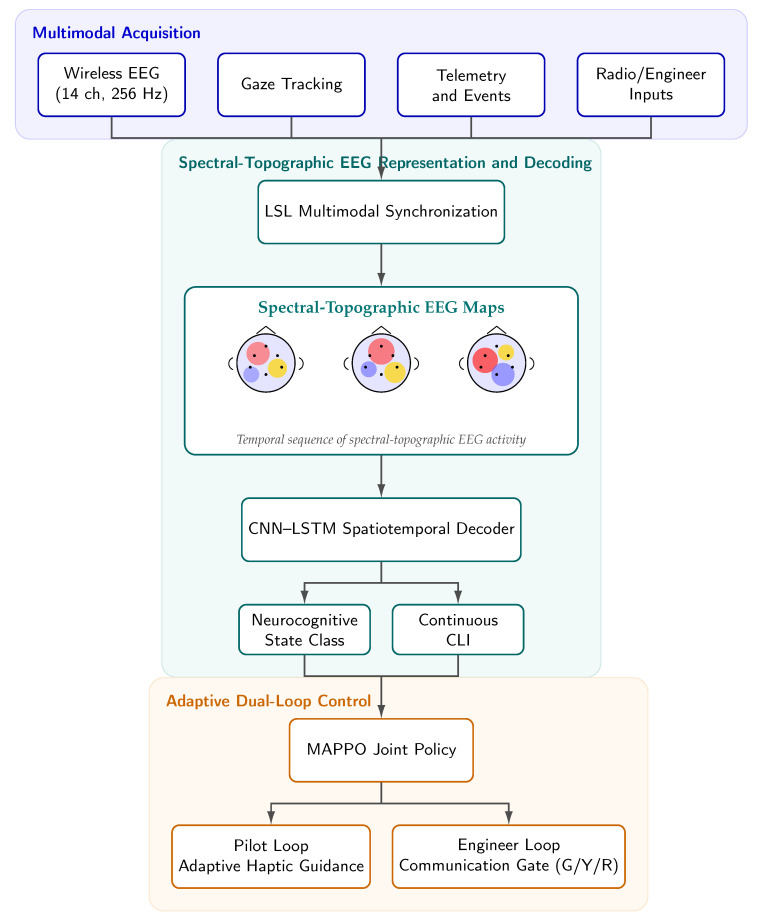
Conceptual overview of the proposed framework. Multimodal streams are synchronized through LSL and transformed into spectral–topographic EEG maps, represented here as time-varying cortical topographies. A CNN–LSTM spatiotemporal decoder extracts neurocognitive state estimates and a continuous cognitive load index, which are then used by a MAPPO controller to regulate both adaptive haptic guidance for the pilot and communication timing for the telemetry engineer. The figure is conceptual and illustrates the representation-and-control pipeline rather than anatomical localization.

In this work, we formulate neuro-adaptation as a real-time problem of spectral-topographic EEG representation learning embedded within a dual-role training environment. We propose a Dual-Loop Neuro-Adaptive Simulation in which dynamic spectral-topographic EEG maps are combined with gaze, telemetry, and communication events to regulate both operator assistance and team-level interaction. Specifically, the framework coordinates two adaptive loops: (i) a pilot loop, in which haptic guidance is modulated to bypass saturated visual and auditory channels [4,5], and (ii) an engineer loop, in which a neuro-informed communication semaphore determines whether radio instructions should be delivered, delayed, or withheld. In this sense, the contribution of the system is not restricted to workload monitoring; rather, it examines whether structured topographic EEG representations can be translated into coordinated control actions in high-pressure human–machine teams under controlled simulation conditions. Figure 1 summarizes the conceptual flow from multimodal acquisition and topographic representation learning to coordinated dual-loop actuation.

### 1.1. From Coordination Failures to Spatially Structured Neurophysiological Representations

In high-stakes operations, communication is often assumed to be inherently beneficial, provided that the content of the message is correct. In practice, however, the timing of communication may be as important as the message itself. Engineers and supervisors may have access to rich external state information, including telemetry, environmental context, and task status, while remaining blind to the operator’s momentary neurocognitive readiness. Under such conditions, additional verbal input can increase interference rather than reduce uncertainty, especially when the operator is already cognitively saturated [1].

This limitation motivates a shift in perspective. Rather than viewing EEG solely as a secondary monitoring channel, we treat it as the basis for constructing spatially organized spectral–topographic representations of ongoing cortical activity. The central question is therefore not only *whether* the operator is overloaded but *how* dynamic spectral–topographic EEG maps can be constructed, decoded, and fused with behavioral data to regulate team interaction. This framing places the problem at the intersection of neurophysiological sensing, topographic representation learning, and adaptive decision support.

### 1.2. From Passive Monitoring to Dual-Loop Neuro-Adaptive Control

Traditional pBCI approaches predominantly focus on passive monitoring or single-user adaptation [3]. Although these systems can estimate workload, vigilance, or attentional shifts, they usually act on the operator alone and do not explicitly regulate the flow of information between interacting human roles. We instead propose a dual-loop neuro-adaptive control architecture in which representation-derived neurocognitive states inform both individual assistance and interpersonal coordination.

In the first loop, the operator receives low-intrusion haptic cues that can deliver urgent information without further loading already congested visual or auditory channels [4,5]. In the second loop, the telemetry engineer receives a neuro-informed communication signal indicating whether intervention is currently appropriate. This transforms “when to speak” into a safety-relevant decision variable rather than an informal human judgment. Importantly, both loops are governed within a shared adaptive framework, enabling coordinated actions that jointly optimize safety, timing, and team efficiency under uncertainty.

### 1.3. Technical Foundations: Spectral–Topographic Decoding, Multimodal Synchronization, and Policy Learning

The proposed framework is enabled by the integration of three technical components within a unified simulation and analysis environment.

First, spatiotemporal neurocognitive decoding is performed through a hybrid CNN–LSTM architecture that processes sequences of spectral–topographic EEG maps. Instead of relying exclusively on scalar channel features, the model captures both the spatial distribution of spectral biomarkers and their temporal evolution across successive windows. This allows the decoder to classify three operationally actionable states—*Channelized Attention*, *Diverted Attention*, and *Surprise/Startle*—while also estimating a continuous Cognitive Load Index (CLI) [6,7,8,9].

Second, multimodal synchronization is implemented using the Lab Streaming Layer (LSL), which aligns EEG, gaze, simulator telemetry, and communication events on a shared temporal basis [10,11]. This temporal alignment is essential because the practical value of cognitive-state decoding depends not only on classification accuracy but also on the ability to relate decoded states to behavioral outcomes and intervention timing within operational windows.

Third, joint adaptive decision-making is implemented through multi-agent proximal policy optimization (MAPPO), which learns a coordinated policy over two coupled outputs: haptic guidance intensity for the pilot and a traffic-light communication gate for the engineer [12,13]. In this pipeline, reinforcement learning does not replace the representation layer; rather, it acts on features derived from spectral–topographic EEG maps to convert decoded neurocognitive states into adaptive control decisions.

### 1.4. Ethical Framing: Neurostate Inference and Privacy by Design

The use of neurophysiological data for real-time adaptation introduces ethical and governance requirements that extend beyond conventional telemetry processing. Neural signals, even when reduced to task-specific indicators, may reveal sensitive attributes related to attention, fatigue, or cognitive vulnerability. For this reason, the proposed framework adopts a privacy-by-design approach aligned with emerging neurorights discussions [14,15,16]. Only minimal, operationally relevant neurostate outputs are exposed to the support role; raw EEG is not repurposed for secondary analysis; and all processing is strictly limited to real-time safety and coordination objectives. This ethical framing is particularly important when neural-state estimates are incorporated into sociotechnical decision systems.

### 1.5. Contributions and Hypotheses

The main contribution of this work is a unified framework for real-time spectral–topographic EEG representation learning and dual-loop neuro-adaptive control in critical human–machine teams. More specifically, the paper contributes:

- A spectral–topographic EEG representation pipeline: dynamic EEG map sequences that support real-time decoding of neurocognitive state beyond conventional channel-wise monitoring alone.
- A real-time decoder for operational neurostates: a CNN–LSTM architecture that outputs both discrete cognitive-state labels (*Channelized Attention*, *Diverted Attention*, and *Surprise/Startle*) and a continuous Cognitive Load Index derived from spectral biomarkers [6,7,8,9].
- A multimodal synchronization layer: LSL-based alignment of EEG, gaze, telemetry, and communication events, enabling temporally reliable attribution between neurostate transitions and intervention outcomes [10,11].
- A dual-output adaptive control policy: a MAPPO-based controller that jointly regulates pilot haptic guidance and engineer communication timing from neurocognitive state estimates derived from topographic EEG representations [12,13].
- A privacy-aware deployment model: a purpose-limited architecture that restricts exposure to minimal neurostate indicators and prevents secondary use of raw EEG beyond operational adaptation [14,15,16].
- A controlled simulation study: an empirical evaluation in a high-fidelity dual-station setting designed to test whether the proposed representation-and-control pipeline yields measurable coordination benefits under time pressure.

We evaluate this framework in a controlled high-fidelity dual-station simulation and test two primary hypotheses:

1. H1 (Communication): Dual-loop neuro-adaptation reduces communication breakdown errors relative to open-loop telemetry, with stronger effects expected during peak-load windows defined by elevated ${\hat{CLI}}_{t}$ and/or critical-failure segments.
2. H2 (Safety/Responsiveness): Neurostate-informed adaptive haptic cues reduce pilot reaction time to critical warnings relative to the open-loop baseline without degrading operational progression.

## 2. Related Work

**Figure 2:**
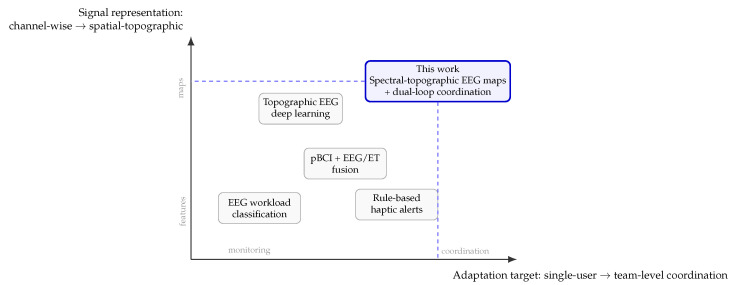
Conceptual positioning of the literature. Most prior work concentrates on either single-user monitoring or rule-based assistance. The present work moves toward team-level coordination while also using spectral–topographic EEG representations rather than purely channel-wise features. The figure is conceptual and summarizes the positioning of the proposed integrated framework relative to adjacent strands of prior work.

Recent literature converges on an important idea: in high-pressure operational environments, cognitive-state estimation is no longer only a human-factors problem but also a problem of *spatiotemporal representation*, *multimodal integration*, and *adaptive decision support*. The most relevant work for the present study spans five lines: (i) EEG-based mental workload and attentional-state decoding, (ii) passive BCI in ecologically valid operational settings, (iii) haptic assistance under sensory contention, (iv) coordinated adaptive control via reinforcement learning, and (v) synchronization and governance for neurodata in real-time systems. Table 1 summarizes these strands, whereas Figure 2 provides a conceptual positioning of the present contribution relative to prior literature.

### 2.1. EEG-Based Mental Workload and Topographic Neurocognitive Representations

Mental workload has traditionally been studied through behavioral and subjective measures, but recent research increasingly relies on neurophysiological signals—especially EEG—to provide continuous and temporally precise estimates of operator state. Recent reviews confirm that frontal theta activity, posterior alpha suppression, and task-dependent cross-frequency patterns remain among the most recurrent EEG correlates of workload, vigilance, and attentional demand [17,18,19]. At the same time, the field still suffers from limited task comparability, inconsistent preprocessing practices, and weak reproducibility across datasets and paradigms [18,36].

For the present study, an important shift in the literature is the move from channel-wise feature engineering toward topology-preserving spatial representations. Instead of treating EEG as a simple multivariate time series, several recent works convert multichannel recordings into topographic maps or spectral–topographic map sequences that preserve electrode geometry while enabling deep spatiotemporal feature extraction [20,21,22]. This trend is methodologically relevant because it reframes EEG analysis as a structured representation-learning problem: neural activity is encoded as a time-varying spatial field that can be decoded using convolutional and hybrid deep-learning architectures.

At the same time, these representations should be interpreted with care. In most wearable-EEG settings, topographic maps are interpolated spatial encodings of scalp-level spectral activity rather than source-resolved neuroimaging. Their methodological value lies in preserving structured spatial relationships for learning and inference, not in providing fine-grained anatomical localization. This distinction is important for situating the present work appropriately.

However, most of this literature remains focused on *offline classification*, *single-user monitoring*, or laboratory tasks such as motor imagery, pain, imagined speech, or generic workload estimation. Much less attention has been paid to operationally actionable state decoding in settings where the inferred neurostate must immediately affect team coordination, support timing, or communication flow.

### 2.2. Passive BCI in Ecologically Valid and Operational Contexts

Passive brain–computer interfaces (pBCIs) infer ongoing cognitive or affective states without requiring deliberate user control, making them attractive for safety-critical domains where explicit interaction is impractical or unsafe [3]. Recent work shows that pBCI can support workload estimation across heterogeneous cognitive tasks [19] and can also be deployed in more realistic settings such as virtual-reality flight training. In particular, van Weelden et al. demonstrated the feasibility of a passive BCI for predicting pilot workload in VR flight scenarios, supporting the operational relevance of portable EEG-based readiness monitoring [23].

A second important trend is the increasing use of multimodal fusion, especially EEG combined with eye tracking. This combination is attractive because EEG captures internal neurocognitive dynamics while gaze and ocular measures describe visual allocation, attentional sampling, and moment-to-moment interaction with the environment. Recent studies in industrial and control-room settings show that EEG + eye-tracking fusion can improve multi-class workload classification relative to single-modality approaches [24], and current reviews highlight this combination as one of the most promising strategies for monitoring workload, vigilance, fatigue, and situational awareness in demanding environments [25].

Yet most pBCI and multimodal studies still end at *state estimation*. They may classify workload or attention, but they do not generally close the loop on *team communication timing*. In other words, the decoded neurostate rarely determines *whether another human should intervene now*. This gap is especially relevant in dual-role operational settings, where the cost of mistimed support may be comparable to the cost of missing support altogether.

### 2.3. Haptic Assistance as a Low-Interference Output Channel

When visual and auditory channels are already congested, haptic feedback becomes a valuable alternative for conveying urgent or directional information with reduced sensory interference. A substantial body of research has shown that vibrotactile feedback can support motor learning, warning delivery, navigation, and hazard awareness with relatively low cognitive overhead [4,5,26]. More recent applied studies continue to refine the design space of vibrotactile alerts. For example, Samsel et al. compared vibrotactile patterns for obstacle-warning systems and showed that pattern design affects both recognition speed and consistency [27]. Likewise, Montano et al. explored mid-air dorsal-hand notifications on the steering wheel as a low-distraction in-vehicle notification channel [28].

Despite these advances, most haptic warning systems remain rule-based. They typically assume that a warning should always be delivered once a threshold is exceeded, regardless of whether the operator is already cognitively saturated. This design logic overlooks the possibility that the same haptic cue may be more or less effective depending on the operator’s neurocognitive readiness and the broader team context. The present work, therefore, differs from prior haptic systems by embedding haptic assistance in a learned policy conditioned on neurocognitive state estimates derived from spectral–topographic EEG representations.

### 2.4. From Human-in-the-Loop RL to Coordinated Multi-Agent Assistance

Adaptive decision-making in dynamic, high-pressure environments often requires policies that are context-sensitive, temporally aware, and jointly optimized across multiple outputs. In this respect, reinforcement learning offers a compelling alternative to fixed thresholds or hand-crafted rules. Recent surveys in human-in-the-loop reinforcement learning emphasize that RL is particularly useful when human state, context, and feedback must be incorporated into the policy loop rather than treated as static exogenous factors [29].

For the present problem, the more relevant subfield is cooperative multi-agent reinforcement learning (MARL). Recent work on centralized training with decentralized execution (CTDE) provides the conceptual foundation for learning coordinated policies that can exploit shared information during training while preserving role-specific execution at runtime [30]. Within this landscape, MAPPO has emerged as one of the strongest and most practical cooperative baselines, with Yu et al. showing that PPO-based multi-agent policies can achieve highly competitive results across multiple cooperative benchmarks [31].

However, there remains a substantial gap between generic MARL benchmarks and human–AI teaming in neuro-adaptive control. Existing work has not, to our knowledge, systematically examined a setting in which one learned policy must simultaneously regulate (i) *pilot-facing haptic assistance* and (ii) *engineer-facing communication timing*, both conditioned on synchronized neurophysiological and behavioral streams. This makes the present formulation meaningfully different from single-agent alert optimization, generic workload adaptation, or standard MARL coordination benchmarks.

### 2.5. Synchronization, Causal Attribution, and Multimodal Timing

A dual-loop neuro-adaptive system is only meaningful if the temporal relationships between communication, neurocognitive state, and behavioral outcome are measured with sufficient precision. Without tight synchronization, it is not possible to determine whether a radio message preceded a startle-related neural pattern, coincided with a workload spike, or followed an already ongoing control degradation.

This requirement makes synchronization infrastructure a central methodological issue rather than a peripheral implementation detail. The Lab Streaming Layer (LSL) has become one of the most widely used frameworks for aligning physiological and behavioral streams in real time, and its recent formal publication in *Imaging Neuroscience* strengthens its status as a rigorous basis for synchronized multimodal recording [32]. For the present setting, LSL is particularly valuable because it supports temporally grounded attribution across EEG, gaze, telemetry, and communication events. This attribution is what ultimately enables the engineer’s semaphore to learn from the consequences of “speak” versus “hold” decisions.

### 2.6. Neurodata Governance, Purpose Limitation, and Mental Privacy

A final line of related work concerns the governance of neurophysiological data in workplace-like or operational environments. The legal and ethical stakes are especially high when brain-derived information is used to infer attentional state, fatigue, or cognitive vulnerability. Within Europe, the GDPR establishes core principles such as *data minimization*, *purpose limitation*, and *storage limitation*, all of which are directly relevant to EEG-based adaptive systems [33]. More recent European guidance has specifically highlighted the emerging category of *neurodata* and the associated risks for privacy, discrimination, and function creep [34,37].

At the international level, neurotechnology governance has also become more explicit. The OECD Recommendation on Responsible Innovation in Neurotechnology provides a foundational policy framework [38], while UNESCO adopted its Recommendation on the Ethics of Neurotechnology in 2025, establishing a new global normative reference for the responsible development and use of neurotechnologies [35]. These developments matter for the present article because they reinforce the need for privacy by design: only minimal neurostate indicators should be exposed to support personnel, and raw EEG should not be repurposed for employability, discipline, or productivity scoring.

### 2.7. Synthesis of the Literature Gap

The literature reviewed above reveals a consistent pattern. Prior studies have advanced EEG-based workload estimation, topographic deep learning, pBCI in operational scenarios, vibrotactile warning design, and cooperative policy learning. However, these advances usually remain separated: workload studies often stop at monitoring, haptic systems remain threshold-based, and MARL benchmarks rarely incorporate synchronized neurophysiological state estimation. As summarized in Table 1, what remains comparatively underexplored is a unified framework that combines:

1. spectral–topographic EEG maps as the neural representation;
2. multimodal synchronization across EEG, gaze, telemetry, and communication;
3. real-time dual-role adaptation, rather than single-user adaptation only;
4. a learned policy that jointly regulates pilot haptics and engineer communication timing.

This combined gap is precisely where the present work is positioned. Figure 2 illustrates this positioning conceptually: compared with prior work, the proposed framework moves simultaneously toward richer spatial representations and toward team-level coordination rather than single-user monitoring. The contribution should therefore be understood not as the first appearance of any individual ingredient, but as an attempt to integrate these ingredients into a controlled simulation framework for dual-role adaptive coordination.

## 3. Materials and Methods

### 3.1. System Overview

The proposed framework is organized as a real-time closed-loop pipeline with five stages: (i) multimodal acquisition; (ii) multimodal synchronization; (iii) construction of spectral–topographic EEG maps; (iv) spatiotemporal neurocognitive decoding; and (v) dual-loop control for coordinated pilot assistance and engineer communication timing. The complete system is designed to preserve temporal correspondence between physiological state, behavioral context, and intervention timing, thereby enabling event-level attribution between engineer speech attempts, pilot neurocognitive state transitions, and downstream safety-relevant outcomes. The present study evaluates this pipeline in a controlled, within-subject, dual-station simulation setting.

Unlike conventional channel-wise EEG processing pipelines, the present methodology treats cortical activity as a sequence of dynamic spatial representations. Specifically, multichannel EEG is transformed into spectral–topographic EEG maps that preserve the relative geometry of the sensor layout and allow the decoder to operate on structured spatial inputs. This design choice is motivated by recent work showing that topographic-map representations improve the learning of spatial–spectral–temporal patterns in EEG compared with purely vectorized channel features [21,22]. The resulting neurocognitive state estimates are then fused with gaze, telemetry, and communication-history descriptors to drive a dual-loop control policy based on MAPPO.

**Figure 3:**
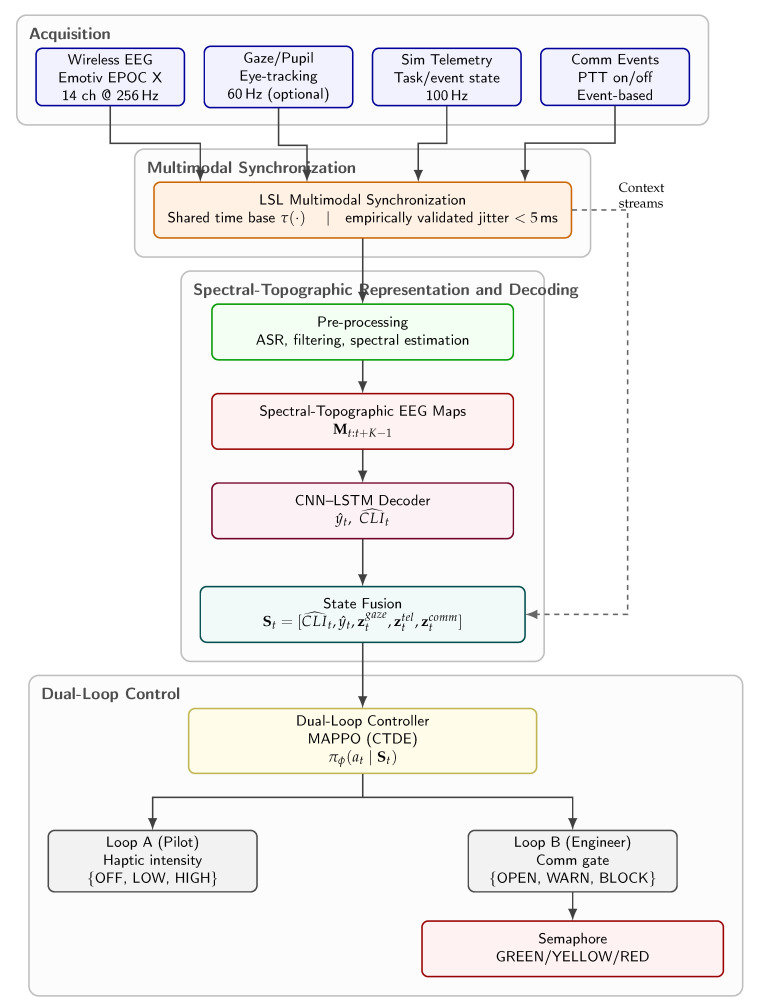
End-to-end methodology. Multimodal streams are synchronized through LSL, transformed into spectral–topographic EEG maps, decoded by a CNN–LSTM model into discrete neurocognitive states and a continuous Cognitive Load Index (CLI), and then fused with contextual variables to drive a dual-loop MAPPO policy. The controller simultaneously regulates pilot-facing haptic assistance and engineer-facing communication timing.

Figure 3 summarizes the end-to-end architecture, Table 2 details the synchronized modalities used in the system, and Table 3 reports the main decoder and controller hyperparameters used in the study.

### 3.2. Multimodal Acquisition and Synchronization

All data streams are synchronized against a shared LSL time base τ(·), following current best practice for multimodal neurobehavioral recording [32]. Each modality is timestamped at acquisition and resampled into windowed observations suitable for real-time inference. Let $x_{t:t+W}^{EEG}$, $x_{t:t+W}^{gaze}$, $x_{t:t+W}^{tel}$, and $x_{t:t+W}^{comm}$ denote synchronized segments collected over a window of length *W*. The multimodal observation is defined as

(1) $$o_{t}=\left[x_{t:t+W}^{EEG},x_{t:t+W}^{gaze},x_{t:t+W}^{tel},x_{t:t+W}^{comm}\right].$$

Online inference uses windows of length W=1 s with stride Δ=100 ms. This configuration provides sufficient temporal granularity for event-level attribution while preserving stable spectral estimates for EEG-derived workload features. The synchronization layer is critical because the system must determine whether a given communication event preceded, coincided with, or followed a neurocognitive shift and its corresponding behavioral consequence.

The synchronization jitter of the Lab Streaming Layer (LSL) was validated empirically by sending a simultaneous hardware pulse (TTL) to the EEG marker stream and to a photodiode placed on the simulator display. Cross-modal latency and jitter were then calculated from the timestamp difference across 1000 test events, confirming a temporal standard deviation below 5 ms. This procedure provides an empirical basis for the sub-frame temporal alignment reported throughout the study.

The use of optional gaze input is motivated by recent multimodal workload literature showing that EEG and eye-tracking provide complementary views of cognitive state, particularly in operational contexts where visual allocation and internal workload jointly shape behaviour [23,24,25,39]. In our pipeline, however, gaze is treated as an auxiliary modality rather than as a hard requirement, so the core system remains deployable with EEG, telemetry, and communication events alone.

### 3.3. EEG Preprocessing

EEG preprocessing was designed to preserve spectral fidelity while remaining compatible with online inference. After acquisition, each 1 s analysis window underwent artifact attenuation, band-pass filtering, notch filtering, and per-window normalization. To attenuate transient high-amplitude artifacts in real time, we used Artifact Subspace Reconstruction (ASR), with the warm-up phase providing the clean reference covariance structure. This choice is consistent with current real-time mobile-EEG practice, where window-wise ICA recomputation is computationally impractical.

The signal was then processed with a zero-phase FIR band-pass filter between 1 Hz and 40 Hz in order to isolate the spectral range of interest while removing slow baseline drifts and high-frequency muscle contamination. To suppress power-line interference without introducing phase distortion, a zero-phase FIR notch filter centered at 50 Hz was applied, with a 2 Hz stopband spanning 49–51 Hz. This configuration is appropriate for the European electrical environment in which the recordings were acquired and is conservative with respect to the present spectral analysis, which focuses on theta, alpha, and beta bands.

### 3.4. Spectral–Topographic EEG Map Construction

Let $X_{t}^{EEG}\in R^{C\times T}$ denote the EEG segment extracted over a window of length *W*, where C=14 channels and *T* is the number of time samples. Each window is projected into a spatial representation defined over the electrode layout. Rather than processing the EEG segment only as a channel vector, we convert it into a stack of spectral–topographic EEG maps that preserves the spatial arrangement of the electrodes and the band-limited distribution of neural activity.

For each band b∈{θ,α,β}, we estimate windowed band power at each electrode and interpolate the resulting values over a regular 2D grid. The canonical bands are defined as θ:4–8 Hz, α:8–12 Hz, and β:13–30 Hz. We also compute frontal alpha asymmetry (FAA) from electrodes F3 and F4 and include it as an auxiliary descriptor, such that the final representation uses B=4 channels (θ, α, β, and FAA).

Electrode positions are first projected from the scalp surface to the 2D map using an azimuthal equidistant projection, thereby preserving relative scalp distances in the planar representation. Spatial interpolation is then performed using biharmonic spline interpolation, yielding smooth continuous maps from the sparse 14-channel layout. The spatial grid resolution is fixed at 32×32 pixels, which provides sufficient granularity for 3×3 convolutional kernels while avoiding unnecessary redundancy for a low-density wearable montage.

The map-construction operator is written as

(2) $$M_{t}=\Phi \;\left(X_{t}^{EEG}\right)\in R^{H\times W_{m}\times B},$$

where $H=W_{m}=32$ and B=4. Over a temporal sequence of K=10 consecutive windows, the decoder receives the tensor

(3) $$M_{t:t+K-1}=\left[M_{t},M_{t+1},\ldots ,M_{t+K-1}\right]\in R^{K\times H\times W_{m}\times B}.$$

Given the 1 s windows and 100 ms stride, this setting provides an effective historical context of approximately 1.9 s, which is long enough to stabilize workload-related dynamics while remaining compatible with low-latency control.

This formulation is consistent with recent EEG work that explicitly transforms multichannel signals into topographic map sequences to capture spatial–spectral–temporal structure with deep models [21,22]. It also improves methodological transparency relative to purely opaque channel-vector pipelines, which is increasingly important in reproducible EEG-based workload research [18,36].

### 3.5. Operational Neurostate Labeling

The decoder was trained to predict three operational neurocognitive classes: Channelized Attention (CA), Diverted Attention (DA), and Surprise/Startle (SU). Because the validity of the decoder depends directly on label quality, class assignment was defined before model training using synchronized task events, behavioral context, and calibration rules.

Windows were labeled as follows:

- CA: windows in which the pilot maintained gaze on the central region of the simulator, executed the primary control task without secondary stimuli, and showed nominal task performance.
- DA: windows corresponding to forced secondary-task engagement, such as auditory n-back interference or lateral panel-management tasks, coinciding with gaze fixation outside the primary task region.
- SU: windows within the 2–3 s interval immediately following unexpected perturbations injected by the system, such as abrupt high-priority visual or auditory alarms or sudden critical failures.

These classes are operational rather than purely descriptive because they are intended to support intervention decisions about when assistance should be intensified, delayed, or withheld. Accordingly, the labels should be interpreted as event-anchored operational states rather than as exhaustive ground-truth descriptions of latent neurocognitive states in a strong neuroscientific sense.

### 3.6. Class Distribution in the Final Labeled Dataset

Because the CA/DA/SU labels were defined from synchronized task events, gaze behavior, and perturbation markers, the final labeled dataset was not expected to be perfectly balanced. This is particularly relevant for the *Surprise/Startle* class, which is intrinsically transient and therefore less frequent than sustained task-engagement states. Under the present protocol, which comprised approximately 40 min of active task time per participant, 1 s windows, and a 100 ms stride, the full cohort yielded a large chronologically structured dataset after ASR-based cleaning and quality control.

The final chronologically partitioned dataset comprised 500,000 valid windows. As shown in Table 4, the class distribution was imbalanced but operationally coherent with the experimental design: *Channelized Attention* (CA) was the majority class with 325,000 windows (65.0%), *Diverted Attention* (DA) accounted for 125,000 windows (25.0%, and *Surprise/Startle* (SU) comprised 50,000 windows (10.0%). This distribution is consistent with the event-anchored nature of the labeling scheme, in which CA reflects sustained primary-task engagement, DA reflects secondary-task interference, and SU captures short-duration perturbation-driven episodes.

This class imbalance should be considered when interpreting decoder performance. For this reason, the Results section reports not only overall accuracy but also balanced accuracy and macro-F1, which are more informative than raw accuracy alone under unequal class frequencies.

### 3.7. Spatiotemporal Neurocognitive Decoder

The neurocognitive decoder is implemented as a hybrid CNN–LSTM model that operates on the sequence of spectral–topographic EEG maps. The convolutional front-end extracts spatial features from each map, while the recurrent back-end models temporal dependencies across consecutive windows. Formally, the decoder learns

(4) $$\left({\hat{y}}_{t},{\hat{CLI}}_{t}\right)=f_{\theta }\;\left(M_{t:t+K-1}\right),\;{\hat{y}}_{t}\in \left\{\mathrm{CA},\mathrm{DA},\mathrm{SU}\right\},$$

where CA denotes *Channelized Attention*, DA denotes *Diverted Attention*, and SU denotes *Surprise/Startle*. In parallel, the network outputs a continuous Cognitive Load Index ${\hat{CLI}}_{t}\in \left[0,1\right]$, which is subsequently used both as a control feature and as a safety-monitoring variable.

The CNN front-end consists of three two-dimensional convolutional layers. The first convolutional layer uses 16 filters with a 3×3 kernel, followed by ReLU activation and 2×2 max pooling. The second convolutional layer uses 32 filters with a 3×3 kernel, followed by ReLU activation and 2×2 max pooling. The third convolutional layer uses 64 filters with a 3×3 kernel and ReLU activation. A spatial dropout of 0.25 is applied after the final pooling stage in order to reduce overfitting.

The flattened spatial feature vectors are then passed to an LSTM with 64 hidden units. A temporal dropout of 0.3 is applied at the recurrent stage. The final latent representation is connected to two task-specific output heads: a three-neuron softmax layer for CA/DA/SU classification and a one-neuron sigmoid layer for continuous CLI estimation.

Training optimizes a joint objective combining categorical cross-entropy for class prediction and mean squared error (MSE) for CLI regression. Model optimization is performed using Adam with learning rate $1\times 10^{-4}$ and weight decay $1\times 10^{-5}$.

The choice of a hybrid spatiotemporal decoder follows recent evidence that topographic map representations benefit from architectures capable of jointly modeling spatial organization and temporal evolution [21,22]. The three-state formulation is operational rather than purely descriptive: it is designed to support intervention decisions about *when* assistance should be intensified, delayed, or withheld.

### 3.8. Chronological Training, Validation, and Test Partitioning

To prevent temporal data leakage, model training and evaluation were performed using a subject-dependent chronological block partition. Windows were not randomly shuffled. Instead, for each participant and session, the earliest 70% of the recording was used for training, the following 10% for validation, and the final continuous 20% for test evaluation. This partitioning reflects the temporal structure of online deployment, in which future windows must remain invisible during training and model selection.

The validation block was used for early stopping and hyperparameter tuning, whereas the final test block served as a held-out evaluation segment for decoder-level reporting. This protocol provides a stricter and more realistic estimate of online generalization than random window shuffling, especially in temporally correlated physiological sequences. At the same time, it should be interpreted as evidence of within-subject temporal generalization rather than subject-independent transfer.

### 3.9. Cognitive Load Index and Participant-Specific Calibration

The framework computes a continuous Cognitive Load Index (CLI), used both as a decoder output and as a control variable. Power spectral density is estimated using Welch’s method, and band powers are aggregated across channels. For each 1 s EEG window, power spectral density was estimated through Welch’s method using a Hann window, segment length $n_{\mathrm{perseg}}=128$ samples (0.5 s), and 50% overlap ($n_{\mathrm{overlap}}=64$ samples). This parameterization yields a frequency resolution of 2 Hz, which is appropriate for the robust extraction of theta, alpha, and beta band activity.

A raw workload score is defined as

(5) $$CLI_{t}^{raw}=\lambda_{1}\;z\;\left({\bar{P}}_{\theta }\left(t\right)\right)-\lambda_{2}\;z\;\left({\bar{P}}_{\alpha }\left(t\right)\right)+\lambda_{3}\;z\;\left({\bar{P}}_{\beta }\left(t\right)\right)+\lambda_{4}\;z\;\left({\mathrm{FAA}}_{t}\right),$$

where z(·) denotes per-participant standardization estimated from warm-up segments, and $\lambda_{i}$ are calibration weights fixed after warm-up. Robust normalization is then applied so that the final index satisfies

(6) $${\hat{CLI}}_{t}\in \left[0,1\right].$$

During a 5 min warm-up phase, the continuous CLI was recorded while each participant performed the baseline task. For each subject, the intra-subject mean μ and standard deviation σ of the CLI were computed. The moderate overload threshold (τ or $\kappa_{yel}$) was then defined as

(7) $$\tau =\kappa_{yel}=\mu +1.5\sigma ,$$

whereas the critical threshold for communication blocking ($\kappa_{red}$) was set to

(8) $$\kappa_{red}=\mu +2.0\sigma .$$

This intra-subject statistical calibration ensures that the policy adapts to each participant’s neurophysiological baseline rather than relying on a fixed population-level threshold.

### 3.10. Multimodal State Fusion for Dual-Loop Control

The output of the neurocognitive decoder is fused with synchronized contextual descriptors to form the control state available to the policy. Let $z_{t}^{tel}$, $z_{t}^{gaze}$, and $z_{t}^{comm}$ denote compact summaries of telemetry, gaze, and recent communication history, respectively. The fused state is defined as

(9) $$S_{t}=\psi \left(o_{t}\right)=\left[{\hat{CLI}}_{t},\;\mathrm{onehot}\left({\hat{y}}_{t}\right),\;z_{t}^{tel},\;z_{t}^{gaze},\;z_{t}^{comm}\right].$$

This state representation is explicitly multimodal and role-agnostic at training time. It exposes the policy to both neurophysiological and task-context information, allowing the controller to learn whether a communication opportunity is safe, mistimed, or unnecessary under the current behavioral and cognitive conditions.

### 3.11. Dual-Loop Control Formulation and MAPPO Training

We formulate control as a cooperative partially observable Markov decision process with centralized training and decentralized execution. The learned policy produces a joint action

(10) $$a_{t}=\langle a_{t}^{pilot},a_{t}^{eng}\rangle ,$$

where

(11) $$a_{t}^{pilot}\in \left\{\mathrm{OFF},\mathrm{LOW},\mathrm{HIGH}\right\},\;a_{t}^{eng}\in \left\{\mathrm{OPEN},\mathrm{WARN},\mathrm{BLOCK}\right\}.$$

The pilot action modulates haptic assistance intensity, whereas the engineer action determines the communication gate. For user-facing interpretability, the engineer gate is mapped to a traffic-light semaphore $g_{t}\in \left\{\mathrm{GREEN},\mathrm{YELLOW},\mathrm{RED}\right\}$. Policy learning is implemented with MAPPO, which is well suited to coordinated multi-output decision-making under shared context [12,31]. This choice also preserves a clean separation between joint training and role-specific runtime outputs.

MAPPO training uses a discount factor γ=0.99, generalized advantage estimation parameter λ=0.95, PPO clipping ratio ϵ=0.2, and entropy coefficient 0.01. The rollout horizon is 2048 steps per environment before each policy update, the minibatch size is 64, and each update is optimized for 10 epochs. Training concludes when the mean episodic reward stabilizes with variation below 5% for 100 consecutive iterations, or after a maximum of $5\times 10^{6}$ environment steps.

### 3.12. Implementation and Runtime Environment

The full neurocognitive decoding and dual-loop control pipeline was implemented in Python 3 using the PyTorch 2.x framework. Real-time inference of the CNN–LSTM decoder and the MAPPO policy (implemented in PyTorch 2.x, PyTorch Foundation, Linux Foundation, San Francisco, CA, USA) was executed on a workstation equipped with an NVIDIA RTX 3090 GPU. Under this configuration, forward-pass times remained below 10 ms, ensuring that the computational cost of model inference remained well below the 100 ms update interval of the online loop and did not constitute a bottleneck for real-time operation.

### 3.13. Engineer Semaphore and Safety Override

To preserve conservative behavior during acute cognitive destabilization, the learned engineer gate is combined with a minimal safety override. Let $g_{t}=\mathrm{map}\left(a_{t}^{eng}\right)$ denote the semaphore derived from the policy action. We impose

(12) $$g_{t}\leftarrow \left\{\begin{array}{cc} \mathrm{RED}, & {\hat{y}}_{t}=\mathrm{SU}\;\mathrm{or}\;{\hat{CLI}}_{t}\geq \kappa_{red},\\ g_{t}, & \mathrm{otherwise}, \end{array}\right.$$

where $\kappa_{red}$ is participant-specific and obtained during the calibration phase. This rule enforces a strict “do not interrupt” condition during surprise/startle episodes or extreme load, while leaving the learned policy free to regulate communication timing under normal and intermediate conditions.

Importantly, the telemetry engineer is exposed only to the semaphore state, not to raw EEG or full decoder outputs. This ensures that the communication interface is driven by purpose-limited neurostate indicators rather than by unrestricted access to neural data.

### 3.14. Reward Design and Constrained Optimization

The instantaneous reward balances safety, task progression, communication hygiene, operator load, and the intrusiveness of haptic assistance:

(13) $$r_{t}=w_{s}\;r_{t}^{safety}+w_{p}\;r_{t}^{progress}+w_{c}\;r_{t}^{comm}-w_{l}\;c_{t}^{load}-w_{h}\;c_{t}^{haptic}.$$

We instantiate the weighting coefficients as

$$w_{s}=10.0,\;w_{p}=1.0,\;w_{c}=0.5,\;w_{l}=0.1,\;w_{h}=0.1,$$

and the auxiliary scaling coefficients as

α=1.0,β=5.0,η=0.1.

This parameterization reflects a safety-first hierarchy: near-miss events and mistimed communication under RED-state conditions are penalized substantially more strongly than task progress is rewarded, while continuous overload and excessive haptic intrusiveness act as lower-weight regularization terms.

We instantiate the reward terms as

(14) $$\begin{array}{ccc} r_{t}^{safety} & = & -I\left[{\mathrm{near}‐\mathrm{miss}}_{t}\right]-\alpha \;\max\left(0,RT_{t}-RT^{★}\right),\;\;\;\;\;\;\;\;\;\;\;\;\;\;\;\;\;\;\;\;\;\;\;\;\;\;\;\;\;\;\;\;\;\;\;\;\;\;\;\;\;\;\;\;\;\;\;\;\;\;\; \end{array}$$

(15) $$\begin{array}{ccc} r_{t}^{progress} & = & \Delta {\mathrm{score}}_{t}\;\mathrm{or}\;-\Delta {\mathrm{time}}_{t},\;\;\;\;\;\;\;\;\;\;\;\;\;\;\;\;\;\;\;\;\;\;\;\;\;\;\;\;\;\;\;\;\;\;\;\;\;\;\;\;\;\;\;\;\;\;\;\;\;\;\;\;\;\;\;\;\;\;\;\;\;\;\;\;\;\;\;\;\;\;\;\; \end{array}$$

(16) $$\begin{array}{cc} r_{t}^{comm} & =+I\left[u_{t}=1\wedge g_{t}=\mathrm{GREEN}\right]-\beta \;I\left[u_{t}=1\wedge g_{t}=\mathrm{RED}\right]-\eta \;I\left[u_{t}=0\wedge g_{t}=\mathrm{GREEN}\right], \end{array}$$

(17) $$\begin{array}{cc} c_{t}^{load} & ={\hat{CLI}}_{t},\;c_{t}^{haptic}=I\left[a_{t}^{pilot}=\mathrm{HIGH}\right],\;\;\;\;\;\;\;\;\;\;\;\;\;\;\;\;\;\;\;\;\;\;\;\;\;\;\;\;\;\;\;\;\;\;\;\;\;\;\;\;\;\;\;\;\;\;\;\;\;\;\;\;\;\;\;\;\;\;\;\;\;\;\;\; \end{array}$$

where $u_{t}$ indicates that the engineer attempted to speak at time *t*, and $RT^{★}$ is the target reaction time for critical hazards.

Because reward shaping alone may still permit undesirable trade-offs, training is additionally cast as a constrained MDP following the safe-RL logic of constrained policy optimization [40]. We define

(18) $$d_{t}^{load}=I\left[{\hat{CLI}}_{t}>\tau \right],\;d_{t}^{cbe}=I\left[u_{t}=1\wedge g_{t}=\mathrm{RED}\right],$$

and the corresponding episodic costs

(19) $$C^{load}\left(\pi \right)=E_{\pi }\left[\frac{1}{T}\sum_{t=0}^{T-1}d_{t}^{load}\right],\;C^{cbe}\left(\pi \right)=E_{\pi }\left[\sum_{t=0}^{T-1}d_{t}^{cbe}\right].$$

The constrained objective is

(20) $$\max_{\pi_{\phi }}\;J\left(\pi_{\phi }\right)=E_{\pi_{\phi }}\left[\sum_{t=0}^{T-1}\gamma^{t}{\tilde{r}}_{t}\right]\;s.t.\;C^{load}\left(\pi_{\phi }\right)\leq \epsilon_{load},\;C^{cbe}\left(\pi_{\phi }\right)\leq \epsilon_{cbe}.$$

In the present study, the constraint budgets were fixed to

$$\epsilon_{load}=0.15,\;\epsilon_{cbe}=0.05,$$

which enforces a conservative limit on overload exposure and on RED-state communication violations at the episode level.

We optimize this objective with a primal–dual Lagrangian:

(21) $$L\left(\phi ,\lambda_{load},\lambda_{cbe}\right)=J\left(\pi_{\phi }\right)-\lambda_{load}\left(C^{load}\left(\pi_{\phi }\right)-\epsilon_{load}\right)-\lambda_{cbe}\left(C^{cbe}\left(\pi_{\phi }\right)-\epsilon_{cbe}\right),$$

with projected multiplier updates after each policy step:

(22) $$\lambda_{i}^{\left(k+1\right)}={\left[\lambda_{i}^{\left(k\right)}+\eta_{\lambda }\left({\hat{C}}^{\;i}-\epsilon_{i}\right)\right]}_{+},\;i\in \left\{\mathrm{load},\mathrm{cbe}\right\},$$

where the dual learning rate was set to

$$\eta_{\lambda }=0.001.$$

This formulation makes explicit that the system prioritizes safety and communication hygiene before marginal gains in task progress.

### 3.15. Experimental Environment, Participants, and Protocol

The experimental setup consisted of a synchronized dual-station simulation environment designed to reproduce continuous pilot control and telemetry-supervision tasks under time pressure. The pilot station comprised a high-fidelity task simulator, force-feedback controls, vibrotactile actuators, and a wireless EEG headset (Emotiv EPOC X, 14 channels, configurable 128/256 Hz acquisition) [41]. Optional gaze tracking was enabled when available. The telemetry station displayed conventional telemetry augmented with the engineer’s semaphore and logged push-to-talk attempts, communication timing, and context markers.

The simulator generated three types of task demands: primary control requirements, secondary-task interference, and perturbation episodes. Secondary task load consisted of auditory *n*-back prompts and lateral panel-management demands, whereas perturbation episodes comprised abrupt high-priority alarms and critical failures. All such events were timestamped within the shared synchronization framework and were subsequently used for CA/DA/SU labeling and peak-load analyses.

A total of N=25 pilot–engineer pairs participated in the study. The experimental protocol comprised four phases: (i) warm-up and calibration, (ii) open-loop baseline runs, (iii) adaptive runs under the pilot-only, engineer-only, and dual-loop conditions, and (iv) post-run debriefing with role-specific analytics.

To mitigate learning and fatigue effects, the order of the four experimental conditions (*Open-loop*, *Pilot-only*, *Engineer-only*, and *Dual-loop*) was counterbalanced across participants using a balanced Latin square design. Each condition lasted 10 min, yielding 40 min of active task time per participant, excluding the initial calibration phase and the 5 min rest periods inserted between consecutive blocks.

Peak-load windows were operationally defined as intervals in which the participant-specific CLI exceeded the overload threshold τ and/or windows located within the 2–3 s period immediately following an unexpected perturbation marker. This definition was used both to quantify overload exposure and to evaluate controller behavior under acute cognitive stress.

The calibration phase served two purposes. First, it normalized decoder outputs at the participant level. Second, it estimated the conservative threshold $\kappa_{red}$ used by the safety override. This participant-specific calibration is particularly important in EEG-based workload systems, where inter-individual variability remains a major challenge for robust generalization [18,23,36]. Accordingly, the present protocol should be interpreted as a controlled feasibility study aimed at within-subject operational evaluation rather than as evidence of subject-independent deployment readiness.

### 3.16. Experimental Conditions

We evaluate four conditions in a within-subject design:

1. Open-loop baseline: no haptic adaptation and no communication gating.
2. Pilot-only: adaptive haptics enabled, while engineer communication remains open-loop.
3. Engineer-only: communication gating enabled, while haptic assistance remains inactive.
4. Dual-loop: full system with both adaptive haptics and engineer semaphore enabled.

Scenario difficulty is manipulated through time-critical hazards and abrupt task perturbations designed to induce peak workload and surprise/startle episodes. This controlled variation enables a more informative comparison of whether the full dual-loop regime improves both safety-related and coordination-related outcomes relative to partial or non-adaptive baselines.

### 3.17. Outcome Variables and Statistical Analysis

The primary coordination metric is the number of Communication Breakdown Errors (CBE), defined as engineer speech attempts made while the semaphore is in the RED state:

(23) $$\mathrm{CBE}=\sum_{t}I\left[u_{t}=1\wedge g_{t}=\mathrm{RED}\right].$$

Primary behavioral outcomes also include pilot reaction time to critical hazards and near-miss counts. Secondary outcomes include overload exposure (time above threshold τ), completion time, task progress, and engineer decision-load proxies such as message-attempt density and communication-timing variance.

Comparisons among the four experimental conditions were analyzed at the session level using one-way repeated-measures ANOVA. Interactions between condition and difficulty were evaluated using factorial ANOVA. For count-like outcomes such as CBE, inference was conducted on per-session aggregated counts at the pilot–engineer pair level rather than on window-level events. When sphericity assumptions were violated, the Greenhouse–Geisser correction was applied. Post hoc paired *t*-tests were adjusted using Bonferroni correction in order to mitigate type-I error inflation under multiple comparisons. In addition to *p*-values, effect sizes were reported as partial eta-squared ($\eta_{p}^{2}$) for ANOVA effects and Cohen’s *d* for paired comparisons, thereby quantifying the magnitude of the observed operational differences.

### 3.18. Ethics and Privacy-by-Design Implementation

The methodology was designed under a privacy-by-design principle. Neural processing is purpose-limited to real-time safety and coordination support; only minimal neurostate outputs are surfaced to the engineer, and raw EEG is excluded from operational dashboards. Data retention is restricted to what is necessary for scientific reproducibility under informed consent, and the semaphore is explicitly not intended for employability, discipline, or productivity scoring.

This boundary is methodological as well as ethical: the control system consumes only the information required to regulate intervention timing and assistance intensity, thereby minimizing the exposure of sensitive neural information while preserving operational utility.

## 4. Results

### 4.1. Decoder Validation on the Held-Out Chronological Test Partition

We first evaluated the neurocognitive decoder independently of the control layer, treating the problem as a spatiotemporal spectral–topographic EEG representation task rather than only as a generic EEG classification problem. All decoder-level metrics reported in this section correspond to the held-out chronological test partition described in Section 3.8, thereby avoiding temporal leakage between training and evaluation. Consistent with the methodological design, these results should be interpreted as evidence of *within-subject chronological generalization* rather than subject-independent transfer.

The CNN–LSTM operating on spectral–topographic EEG maps achieved an overall accuracy of 93.6% across the three operational neurocognitive classes—*Channelized Attention* (CA), *Diverted Attention* (DA), and *Surprise/Startle* (SU). Importantly, the *Surprise/Startle* class, which is the most safety-critical category for communication blocking, reached a recall of 0.91. These results indicate that the downstream controller is driven by a discriminative and operationally meaningful neurostate signal rather than by a weak surrogate marker.

**Figure 4:**
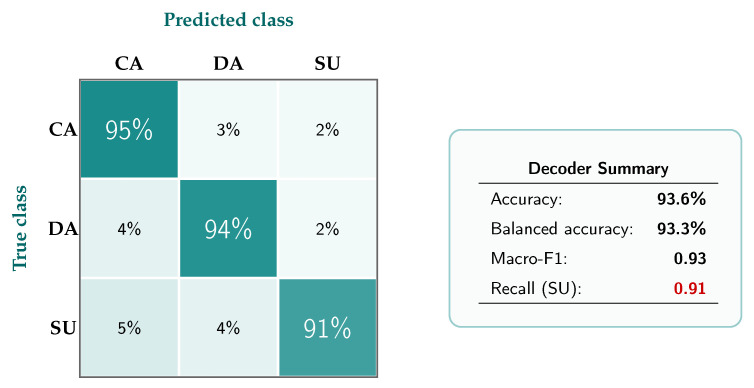
Normalized confusion matrix of the CNN–LSTM decoder operating on spectral–topographic EEG maps. The diagonal concentration confirms high class separability, while the recall of the safety-critical *Surprise/Startle* class remains high enough for reliable communication blocking.

To better reflect class-balanced performance, Table 5 additionally reports balanced accuracy and macro-F1. In addition, based on the normalized class-wise recalls shown in Figure 4, balanced accuracy reached 93.3%. Macro-F1 reached 0.93, indicating that performance remained strong across all operational classes and was not dominated by a single state.

### 4.2. Class-Wise Confusion Structure

Figure 4 reports the normalized confusion matrix of the decoder. Most of the probability mass is concentrated on the diagonal, confirming high class separability across CA, DA, and SU. The most challenging category remains *Surprise/Startle*, as expected for abrupt neurocognitive transitions, yet its recall remains sufficiently high to support reliable safety-oriented communication blocking. Operationally, this confusion structure is favorable because the safety-critical SU state is only rarely diluted into non-critical categories.

### 4.3. Representation Ablation: Why Spectral–Topographic EEG Maps Matter

To isolate the contribution of the proposed input representation, we compared three decoder configurations: (i) raw channel-wise EEG windows, (ii) spectral feature vectors composed of band-power summaries and frontal alpha asymmetry, and (iii) the proposed spectral–topographic EEG maps. All three variants were evaluated under the same chronological subject-dependent partitioning protocol.

This ablation is important because the central methodological claim of the paper is not only that EEG can decode operational neurostates but also that a spatially structured representation improves discrimination and real-time usability relative to non-spatial alternatives.

As shown in Table 6, the proposed spectral–topographic representation yielded the best overall performance. Raw channel-wise EEG windows achieved the lowest performance, with an accuracy of 84.2%, balanced accuracy of 83.8%, and macro-F1 of 0.84, confirming that the lack of an explicit spatial prior limits discriminative power. Spectral feature vectors improved performance to 89.1%, 88.7%, and 0.89, respectively, indicating that band-wise feature engineering captures meaningful workload-related information but still collapses the spatial organization of cortical activity. By contrast, the proposed spectral–topographic EEG maps achieved 93.6% accuracy, 93.3% balanced accuracy, and 0.93 macro-F1, confirming that preserving the spatial arrangement of electrode activity provides the most effective representation for the present formulation.

### 4.4. Temporal Stability of Inference and Runtime Characteristics

Beyond raw discrimination performance, a neuro-adaptive pipeline intended for online deployment must satisfy two additional requirements: temporal stability and real-time throughput. Temporal stability determines whether adjacent predictions remain sufficiently consistent to support human-facing control signals, while throughput determines whether the complete decoding-and-control loop can operate within the inference budget imposed by the stride Δ=100 ms.

In the current system, adjacent-window class agreement reached 94.5%, showing that consecutive predictions were highly stable over time and that the interface was unlikely to oscillate erratically between states. Likewise, the median absolute difference of the continuous CLI between consecutive windows was only 0.025, confirming that the normalized cognitive load trajectory was smooth and not dominated by high-frequency noise. These values indicate that the neurostate estimates are not only accurate but also temporally coherent enough for real-time adaptive control.

From the computational perspective, end-to-end latency from EEG window completion to actuation remained below 50 ms, which is comfortably below the 100 ms stride of the online loop. This implies a sustained online throughput above 20 windows/s for the full pipeline. Table 7 summarizes these temporal-stability and runtime metrics for the online decoder and full dual-loop pipeline.

### 4.5. Controller-Level Evaluation

Once the decoder had been validated as a reliable spatiotemporal neurocognitive component, we evaluated the complete dual-loop control framework under the four experimental conditions described in Section 3.16: open-loop baseline, pilot-only, engineer-only, and dual-loop. All controller-level comparisons were performed at the session level across the N=25 pilot–engineer pairs. At this stage, the central question is no longer whether neurostate can be decoded but whether the decoded CLI and operational class labels improve coordinated pilot–engineer behavior.

### 4.6. Pilot Responsiveness to Time-Critical Hazards

Under high visual load, the open-loop baseline yielded a reaction time (RT) of 487±156 ms. In the pilot-only condition, RT decreased to 219±72 ms, whereas the engineer-only condition remained close to baseline at 482±150 ms. In the full dual-loop condition, RT remained low at 231±77 ms. Relative to the open-loop baseline, this corresponds to an approximate reduction of 55% in the pilot-only condition and 53% in the dual-loop condition.

These results show that the complete controller preserves most of the haptic benefit while simultaneously incorporating communication regulation, confirming that dual-loop control improves time-critical responsiveness without relying exclusively on the auditory channel.

### 4.7. CLI Reduction, Overload Exposure, and Operational Efficiency

The controller also improved the continuous cognitive-state trajectory as measured through overload exposure derived from the CLI. In the open-loop baseline, overload exposure reached 14.2±4.3% of the session. This value fell to 10.5±3.8% in the pilot-only condition, 13.8±4.1% in the engineer-only condition, and to 7.6±2.8% in the full dual-loop condition. Relative to open-loop, the dual-loop controller, therefore, reduced overload exposure by approximately 46.5%.

At the task level, completion time improved modestly by 3.2% relative to open-loop, and near-miss counts decreased by 12% in high-difficulty scenarios. This pattern is important because it shows that delaying or suppressing mistimed engineer interventions did not impair operational tempo; rather, the dual-loop policy improved safety margins while preserving realistic task progression.

### 4.8. Communication Breakdown Errors (CBE) and Team Coordination

The main coordination outcome was the number of Communication Breakdown Errors (CBE), defined as engineer speech attempts made while the semaphore indicated a RED state. In the open-loop condition, engineers committed 4.1±1.3 CBE per session. This value decreased to 3.8±1.2 in the pilot-only condition, 3.1±1.1 in the engineer-only condition, and 2.7±0.9 in the full dual-loop configuration.

Relative to open-loop telemetry, the full dual-loop controller, therefore, reduced CBE by approximately 31%. This contrast was statistically significant (p<0.001), with a large paired effect size (d=1.85). The estimated mean reduction was 1.4 CBE per session (95% CI [1.09,1.71]), indicating that the engineer-side gating policy produced a substantial coordination benefit beyond the partial improvements observed in the single-loop conditions.

Importantly, this benefit was not uniform across the entire session. When the analysis was restricted to peak-load windows—that is, intervals satisfying the operational criterion defined in Section 3.15—the reduction in interruption errors reached up to 73%. For this conditional analysis, the peak-load subset comprised 50,000 windows associated with acute surprise/startle episodes, corresponding to 10.0% of the final chronologically partitioned dataset. Within this high-risk subset, the mean pilot reaction time was 250.00 ms, with an approximate 95% confidence interval of [233.49, 266.51] ms. This indicates that the dual-loop controller preserved a stable low-latency response profile even during the most demanding operational intervals. Accordingly, the reported 73% reduction should be interpreted as a *conditional effect* on a predefined high-risk subset rather than as a session-wide average effect.

### 4.9. Inferential Summary Across Experimental Conditions

**Figure 5:**
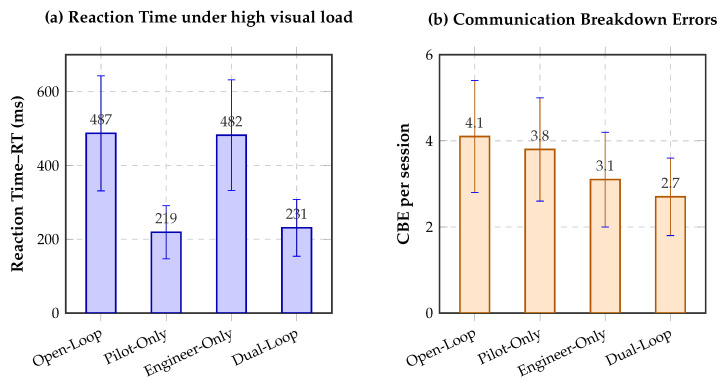
Controller-level outcomes separated from decoder validation. (**a**) Reaction time (RT) under high visual load. (**b**) Communication Breakdown Errors (CBE) per session. Bars show mean values and error bars denote standard deviation across the N=25 pilot–engineer pairs.

Table 8 summarizes the principal inferential results for the controller-level outcomes under the repeated-measures design. Together with the descriptive statistics reported above, these tests confirm that the dual-loop condition consistently outperformed the open-loop baseline on the main coordination and workload-related outcomes. Figure 5 then visualizes the two principal controller-level outcomes, reaction time and communication breakdown errors.

**Figure 6:**
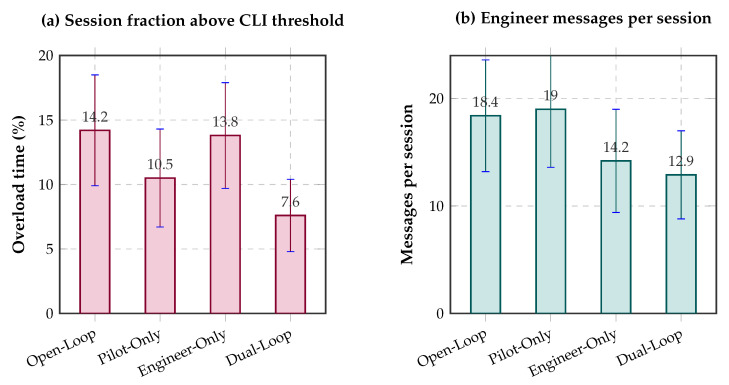
Controller-level burden and communication outcomes. (**a**) Overload exposure, defined as the fraction of each session where ${\hat{CLI}}_{t}>\tau$. (**b**) Engineer messages per session. Bars show mean values and error bars denote standard deviation across the N=25 pilot–engineer pairs.

Figure 6 complements these results by visualizing overload exposure and engineer message volume across the same four controller conditions.

### 4.10. Joint Outcome Summary Across Conditions

Table 9 summarizes the joint trade-off across responsiveness, communication timing, overload exposure, and engineer message volume. The dual-loop controller achieved the most balanced operational profile: it preserved low RT, produced the fewest CBE, reduced overload exposure, and lowered engineer message volume relative to the open-loop baseline.

### 4.11. Latency, Synchronization, and Real-Time Feasibility

The control-level improvements must be interpreted together with the timing guarantees of the acquisition and inference stack. LSL synchronization jitter remained below 5 ms across modality pairs, enabling event-level attribution between engineer speech attempts, decoded neurostate transitions, and pilot behavioral responses. End-to-end latency from EEG window completion to actuation remained below 50 ms, satisfying the real-time requirements of the studied warning and communication horizons. These timing results confirm that the full chain—from multimodal synchronization to spectral–topographic representation, neurostate decoding, and dual-loop actuation—operates within the temporal budget required by the task.

### 4.12. Interpretation of the Combined Results

Taken together, the results support a two-level interpretation. At the decoder level, the CNN–LSTM operating on spectral–topographic EEG maps provides accurate, class-balanced, and real-time-compatible neurocognitive-state estimation. The ablation analysis further shows that this performance depends not only on the choice of model but also on the representation itself: raw channel-wise windows and compact spectral features remain competitive, but both are outperformed by the spatially structured topographic formulation. In addition, the high adjacent-window agreement and low inter-window CLI variation demonstrate that the decoder produces temporally stable outputs suitable for continuous interaction.

At the controller level, these neurostate estimates are successfully translated into actionable coordination benefits: faster pilot responses, lower CLI overload exposure, and fewer Communication Breakdown Errors. This separation is central to the logic of the paper. The contribution is not merely that a classifier reaches high accuracy, nor merely that a controller improves operational outcomes. Rather, the core result is that a spectral–topographic EEG representation can be transformed into a dual-loop control policy that improves both individual responsiveness and team communication timing in critical environments. Because the study is simulation-based and subject-dependent, these findings should be interpreted as evidence of controlled feasibility rather than definitive proof of field-level generalization.

## 5. Discussion

### 5.1. From Spectral–Topographic EEG Representations to Dual-Loop Coordination

The central implication of the present study is that neuro-adaptation in critical environments should not be framed exclusively as an individual monitoring problem but as a team-level coordination problem supported by spectral–topographic EEG representations. The results show that the contribution of the system emerges at two distinct yet interconnected levels. First, the decoder operating on spectral–topographic EEG maps provides accurate, class-balanced, and temporally stable estimates of operational neurocognitive state under a held-out chronological evaluation protocol. Second, these neurostate estimates are translated into a dual-loop control policy that improves both pilot responsiveness and communication timing.

This distinction is important because many prior systems stop at cognitive-state estimation. In contrast, the present framework suggests that the practical value of neurocognitive-state inference increases substantially when it is embedded into a control architecture that regulates *when* support should be delivered and *through which channel*. In this sense, the main advance is not merely the decoding of workload-related states but the conversion of those states into coordinated pilot–engineer adaptation under temporally aligned multimodal evidence.

The decoder was trained and evaluated on an imbalanced but operationally realistic dataset, with CA as the majority class, DA as an intermediate class, and SU as the minority transient class. This makes the strong balanced accuracy and macro-F1 values particularly relevant, since performance was not driven only by the dominant class.

### 5.2. Why Spectral–Topographic EEG Maps Matter

A second major implication concerns representation. The ablation analysis shows that spectral–topographic EEG maps outperform both raw channel-wise EEG windows and compact spectral feature vectors. This result supports the methodological premise of the paper: representing EEG as a sequence of spatially organized maps is not only a reformulation of the signal but also a functionally meaningful design choice.

From a representation-learning perspective, this matters because cortical activity is not treated as an unordered set of channels but as a dynamic spatial field whose temporal evolution can be learned through hybrid convolutional–recurrent models. The superiority of the topographic representation suggests that preserving electrode geometry helps the decoder capture neurocognitive structure that is partially lost when the same data are collapsed into feature vectors. The main contribution, therefore, lies not only in classification accuracy but in showing that a spatially structured topographic representation improves real-time operational inference.

At the same time, this claim should be interpreted with precision. The proposed maps are *interpolated spectral–topographic representations* derived from a 14-channel wearable EEG system; they should not be conflated with high-density source-resolved neuroimaging. Their value lies in providing an operationally useful spatial encoding of band-limited activity under realistic sensing constraints, rather than in offering fine-grained anatomical localization. This distinction is important for methodological clarity and for positioning the contribution appropriately within the literature.

Equally important, the temporal-stability results indicate that the decoder is usable in practice rather than only in offline evaluation. The high adjacent-window agreement and the low median absolute variation of the CLI suggest that the neurostate estimates form a stable control signal, thereby reducing the risk of erratic interface oscillations or spurious communication gating. This stability is especially relevant in real-time decision-support settings, where over-reactive state estimation may be as problematic as poor discrimination.

### 5.3. Decoder-Level Gains and Controller-Level Gains Should Not Be Confused

The results also clarify that decoder-level success and controller-level success are related but not interchangeable. A highly accurate decoder is necessary but not sufficient for operational benefit. The controller must still convert the decoded CLI and class labels into interventions that are both timely and context-sensitive. This is why the present manuscript separates the validation of the spectral–topographic EEG map decoder from the evaluation of the dual-loop control policy.

At the decoder level, the system demonstrates that neurocognitive states can be estimated with strong accuracy, balanced performance, and real-time-compatible latency under a chronological held-out evaluation protocol. At the controller level, the system demonstrates that these estimates can be converted into tangible operational improvements, including reduced Communication Breakdown Errors (CBE), reduced overload exposure, and faster pilot responses under visual saturation. This separation is methodologically important because it avoids a common ambiguity in human–AI systems: improvements in downstream behavior should not be attributed to the classifier alone when they actually depend on the structure of the control policy.

### 5.4. From Individual Assistance to Sociotechnical Optimization

The controller results suggest that neuro-adaptation should be interpreted as a form of sociotechnical optimization rather than as a single-user augmentation strategy. Pilot-only assistance substantially improves reaction time but cannot eliminate the main team-level failure mode: mistimed verbal interventions during periods of cognitive saturation. Conversely, engineer-side gating reduces CBE but does not directly improve the pilot’s sensorimotor responsiveness to hazards. The strongest profile emerges only when both loops are coordinated.

This finding is theoretically relevant because it suggests that human–AI teaming benefits arise when the AI mediates coordination, not merely perception. In the present setting, the AI does not simply warn the pilot or silence the engineer; it regulates the timing and channel distribution of support based on the evolving neurocognitive state of the operator. The fact that the largest CBE reductions occur during peak-load windows is particularly important: it suggests that the controller is not improving outcomes through indiscriminate suppression of communication but through selective timing control when interference is most harmful.

At the same time, this interpretation should remain appropriately bounded. The dual-loop configuration also reduces overall engineer message volume, which means that part of the gain is necessarily associated with more selective communication behavior. The relevant point is therefore not that the system preserves all messages, but that it improves the timing and density of interventions while maintaining operational progress and improving hazard responsiveness.

### 5.5. Role of Multimodal Synchronization in Causal Interpretation

Another important point is that the operational meaning of the results depends directly on multimodal synchronization. The contribution of the system is not only that EEG, telemetry, gaze, and communication are all recorded, but also that they are aligned with sufficient temporal precision to support causal interpretation of intervention timing. Without such synchronization, it would not be possible to determine whether a communication attempt preceded a rise in CLI, coincided with a *Surprise/Startle* episode, or followed an already ongoing control deviation.

This means that multimodal synchronization is not a technical convenience but a methodological prerequisite for the entire dual-loop argument. The reduction in CBE and the interpretation of RED-state interventions only become meaningful because the system can temporally attribute communication attempts, neurocognitive transitions, and behavioral responses to the same event structure.

### 5.6. Operational Labels, Neurocognitive Meaning, and Interpretation Boundaries

The present results should also be interpreted in light of how the neurocognitive classes were defined. The labels *Channelized Attention*, *Diverted Attention*, and *Surprise/Startle* were operationalized from synchronized task events, gaze behavior, and perturbation markers, rather than from an external gold-standard measure of latent cognitive state. This design is appropriate for an intervention-oriented control framework because the controller ultimately requires *operationally actionable* state distinctions. However, it also imposes an interpretive boundary: the reported accuracy should be read as performance relative to an event-anchored operational labeling scheme, not as a claim of exhaustive access to underlying mental states in a strong neuroscientific sense.

This distinction matters because the classifier is optimized for timing-sensitive adaptation in a simulated operational task, not for universal neurocognitive taxonomy. In practice, this is sufficient for the present application; nevertheless, future work should examine whether the same class structure remains stable across broader task families, different perturbation types, and alternative labeling protocols, including external raters or additional physiological criteria.

### 5.7. Neural Privacy, Purpose Limitation, and the Limits of Operational Inference

The safety benefits of neurostate estimation also introduce an ethical tension. Unlike conventional telemetry, neural signals can reveal aspects of internal cognitive condition that extend beyond the immediate operational task. This creates a familiar but unresolved paradox: the system is most useful when it has access to meaningful neurocognitive indicators, yet those same indicators can become problematic if repurposed for surveillance, employability judgments, or performance profiling.

The present framework addresses this tension through privacy-by-design and purpose limitation. Only low-bandwidth operational outputs are surfaced to the engineer; raw EEG and high-dimensional neural descriptors are excluded from the operational interface; and the dual-loop controller uses only the information necessary to regulate haptic intensity and communication timing. This is not merely an ethical add-on but part of the methodological logic of the system. By restricting the exposed output to a semaphore-like decision layer, the framework preserves the operational benefit of neuro-adaptation while minimizing the risk of interpretive overreach.

From a governance standpoint, this suggests that neuro-adaptive systems in workplace-like environments should be evaluated not only by accuracy and latency but also by the narrowness of the inference pathway between raw neurodata and operational action. In other words, the question is not only whether the model works but also whether it reveals more than is necessary for the task it is intended to support.

### 5.8. Human Agency and the Risk of Automation Overreach

A related concern is the possibility of *automation overreach*. If the engineer becomes overly dependent on the semaphore, or if the system blocks a message that would have been genuinely safety-critical, responsibility boundaries may become blurred. The present design partially mitigates this risk by using a three-level communication gate rather than a binary open/close policy. The YELLOW state preserves a space for delay, prioritization, and human judgment, rather than enforcing hard suppression in all non-GREEN situations.

This design choice reinforces the intended role of the AI as a coordination mediator rather than a decision replacement mechanism. The dual-loop controller is meant to reduce mistimed interventions and channel contention, not to eliminate human discretion. Future work should therefore examine not only objective performance but also trust calibration, override behavior, and the long-term effects of relying on neuro-informed gating in repeated operational settings.

### 5.9. Limitations

Several limitations should be acknowledged. First, the study is conducted in a high-fidelity simulation environment rather than in live deployment. Although this enables controlled synchronization and precise attribution of communication effects, real-world settings introduce additional motion artifacts, stressors, and contextual variability that may challenge both decoder robustness and policy transfer. The present results should therefore be interpreted as evidence of controlled feasibility rather than definitive field validation.

Second, the sample size is moderate (N=25 pilot–engineer pairs). Although the within-subject design increases sensitivity to condition-level effects, this sample still limits the precision with which inter-individual variability, rare failure cases, and subgroup-specific patterns can be characterized. In particular, the strong gains reported in some outcomes should be interpreted with appropriate caution until replicated in larger cohorts.

Third, the current EEG setup is based on a 14-channel wireless device, which is practical for ecologically valid deployment but necessarily provides lower spatial resolution than laboratory-grade systems. The reported gains, therefore, demonstrate feasibility under realistic sensing constraints, but not the upper bound of representation fidelity. Relatedly, the current spectral–topographic EEG maps are constructed from a restricted spectral set and a relatively compact spatial layout. Richer spatial interpolation schemes, denser head models, or uncertainty-aware map representations may further improve discrimination.

Fourth, although the chronological block partition used for decoder evaluation provides a stricter estimate of online generalization than random shuffling, it remains a subject-dependent protocol. The present results, therefore, support within-subject temporal generalization more directly than cross-subject transfer. Additional evaluation under subject-independent or cross-domain settings remains necessary.

Fifth, the current controller focuses on communication gating and haptic intensity modulation; more expressive policies, such as message prioritization, semantic compression, or adaptive delay scheduling, remain outside the present scope. Sixth, although the temporal-stability metrics support the online usability of the decoder, longitudinal robustness under fatigue, drift, or user adaptation still requires further study.

Finally, the current evidence for real-time feasibility remains tied to the present hardware and simulation setup. Although end-to-end latency and synchronization performance are compatible with online use in this environment, broader engineering feasibility across other platforms and operational settings remains to be demonstrated.

### 5.10. Further Research Directions

The results suggest several directions for future work. At the representation level, one natural extension is to explore more advanced forms of spectral–topographic EEG representation learning, including uncertainty-aware topographic sequences, attention-based spatial encoders, or multimodal map fusion with gaze heatmaps. At the control level, future work could expand the current dual-loop control framework into richer forms of team assistance, such as message ranking, adaptive queueing, or semantically compressed engineer feedback.

A second direction concerns transfer and personalization. Because inter-individual variability remains a major challenge in EEG-based workload decoding, future studies should examine subject-adaptive fine-tuning, domain adaptation across simulators, and robustness under prolonged sessions. A third direction concerns governance: neuro-adaptive teaming systems should be accompanied by explicit rules for retention, escalation, override authority, and independent oversight, especially in domains where consent may be structurally pressured.

A fourth direction concerns stronger validation of the neurocognitive labels themselves. Future studies could combine event-grounded labels with expert annotation, additional physiological markers, or cross-session agreement analyses in order to better quantify how stable and transferable the CA/DA/SU taxonomy is across contexts and participants.

A fifth direction concerns stronger statistical and operational validation. Future studies should complement aggregate performance summaries with exact inferential outputs, confidence intervals for key reductions, and clearer reporting of peak load episode counts, thereby making the controller-level evidence easier to audit and reproduce.

### 5.11. Concluding Perspective

Overall, the discussion supports a broader interpretation of the study. The present contribution is not simply a passive BCI, nor merely a reinforcement-learning controller. Rather, it is a demonstration that spectral–topographic EEG representations, when combined with multimodal synchronization and dual-loop control, can support a new class of neuro-adaptive coordination systems in which neurocognitive-state estimates improve both individual responsiveness and team communication timing. This combination of representation, synchronization, and control is what gives the framework its methodological and practical significance, while the present results position that contribution most appropriately as a controlled and promising step toward future field-valid neuro-adaptive teamwork systems.

## 6. Conclusions and Future Directions

This paper presented a Dual-Loop Neuro-Adaptive Simulation for dual-role coordination in high-pressure operational settings, showing how spectral–topographic EEG representations can be translated into actionable team-level support. The proposed framework combines three elements within a unified real-time pipeline: (i) multimodal synchronization through LSL with cross-modal jitter below 5 ms, (ii) a CNN–LSTM decoder operating on spectral–topographic EEG maps to estimate both discrete operational neurocognitive states and a continuous Cognitive Load Index (CLI), and (iii) a dual-loop control policy based on MAPPO that jointly regulates pilot-facing haptic guidance and engineer-facing communication timing.

The results support two complementary conclusions. At the decoder level, the proposed representation achieved strong neurostate discrimination, with 93.6% overall accuracy, 93.3% balanced accuracy, a macro-F1 of 0.93, and high recall for the safety-critical *Surprise/Startle* class. The ablation analysis further showed that spectral–topographic EEG maps outperform both raw channel-wise EEG windows and compact spectral feature vectors, confirming that preserving spatial organization is not merely a representational preference but a functionally important design choice. In addition, the decoder remained temporally stable across adjacent windows and operated within a real-time budget compatible with online deployment.

At the controller level, the decoded neurocognitive states were successfully translated into measurable operational benefits across the four evaluated conditions. Neuro-adaptive haptics substantially improved pilot reaction time under visual saturation, while communication gating reduced Communication Breakdown Errors (CBE) relative to open-loop telemetry. The full dual-loop control framework produced the most balanced overall profile, combining faster hazard responsiveness, lower CLI overload exposure, fewer interruption errors, and lower engineer message volume without compromising realistic task progression. Particularly important is the fact that the largest gains were observed during peak-load windows, where mistimed interventions are most harmful. This suggests that the value of the framework lies not simply in suppressing communication but in regulating *when* communication should occur.

Taken together, these findings support the central claim of the manuscript: neuro-adaptation in critical environments should not be treated only as an individual assistance problem but as a sociotechnical coordination problem. More specifically, the study demonstrates that spectral–topographic EEG representations, when coupled with multimodal synchronization and dual-loop control, can improve both individual responsiveness and team communication timing in safety-critical settings. This is the main methodological contribution of the paper.

At the same time, the contribution should be interpreted with appropriate methodological precision. The proposed maps are interpolated topographic representations derived from a wearable 14-channel EEG system, not high-density source-resolved neuroimaging. Their contribution lies in providing an operationally useful spatial encoding of band-limited cortical activity under realistic sensing constraints. In this sense, the study does not claim fine-grained anatomical inference; rather, it shows that structured topographic EEG representations are sufficient to support accurate neurostate decoding and effective adaptive coordination in a real-time human–AI loop.

A second point of caution concerns generalization. Decoder-level evaluation was conducted under a chronologically held-out subject-dependent protocol, which provides a realistic estimate of within-subject online deployment but does not by itself establish subject-independent transfer. Likewise, the controller results were obtained in a high-fidelity simulation rather than in live field deployment. The present study should therefore be understood as evidence of controlled feasibility and operational promise, rather than as definitive proof of cross-context robustness.

### Future Directions

Several directions follow naturally from the present work.

- Field transfer and robustness. Future studies should validate the framework under real deployment conditions, including motion artifacts, vibration, prolonged workload, and environmental noise. This will require artifact-aware decoding, domain adaptation, and robustness analysis across longer operational sessions.
- Richer spectral–topographic EEG representations. Although the present work uses a compact representation derived from a 14-channel system, future research could investigate denser spatial interpolation schemes, uncertainty-aware map construction, attention-based spatial encoders, and multimodal map fusion with gaze-derived representations.
- Personalized and online adaptation. The current pipeline includes participant-specific calibration, but future work should examine safe online adaptation of CLI thresholds, subject-adaptive decoder tuning, and policy personalization under safety-first constraints, especially in the presence of fatigue, learning, or drift.
- Communication support beyond gating. The present system deliberately uses a minimal semaphore for privacy and interpretability. Future extensions could include message prioritization, adaptive delay queues, compressed telemetry summaries, or structured language templates, provided these are evaluated against both cognitive-load reduction and privacy-by-design requirements.
- Cross-subject and cross-domain generalization. The dual-loop principle is likely to extend beyond pilot–engineer teams to other safety-critical settings, such as surgeon–assistant coordination, emergency response command structures, or industrial control-room operations. Future work should therefore examine both subject-independent transfer and domain transfer to determine which elements of the decoder and controller generalize and which require domain-specific adaptation.
- Governance and deployment protocols. Future deployment should be accompanied by explicit safeguards for purpose limitation, retention boundaries, auditability, human override, and independent oversight so that neurostate outputs remain safety instruments rather than employability or productivity signals.
- Stronger validation of operational labels. Because the CA/DA/SU taxonomy is event-grounded and operationally defined, future work should examine its stability across sessions, participants, and task domains and should compare it against additional physiological criteria, expert annotation, or cross-modal agreement measures.
- Stronger inferential reporting and reproducibility. Future studies should pair aggregate performance summaries with exact inferential outputs, confidence intervals for key reductions, clearer reporting of peak-load episode counts, and, where possible, reproducible analysis pipelines that facilitate independent verification.

The present study establishes that spectral–topographic EEG representations are not only a viable basis for real-time neurocognitive-state decoding but also a practical foundation for dual-loop neuro-adaptive coordination. By linking representation, synchronization, and control within a single operational framework, the work opens a path toward a new class of human–AI teaming systems for critical environments. At its strongest, the present contribution should be understood as a controlled and methodologically explicit demonstration that structured wearable-EEG representations can support adaptive coordination policies with promising operational value, while also defining a clear agenda for the stronger cross-subject, field-level, and governance-aware validation that remains necessary.

Detailed mathematical formulations of the multi-agent POMDP and the Cognitive Load Index are provided in Appendices Appendix A and Appendix B.

## Figures and Tables

**Table 1:** Summary of the most relevant related-work strands and the gap addressed in this paper.

Research Line	Representative Studies	Main Contribution	Remaining Gap
EEG workload estimation	[17,18,19]	Continuous estimation of workload and attentional demand from EEG biomarkers	Limited task transfer, weak reproducibility, and mostly single-user state estimation
Topographic EEG deep learning	[20,21,22]	Topology-preserving EEG map representations for spatiotemporal decoding	Mostly offline or non-operational tasks; little direct coupling to team-level intervention
Operational pBCI and multimodal fusion	[23,24,25]	Feasibility of pBCI in realistic tasks and improved workload inference via EEG + eye tracking	Fusion improves monitoring, but rarely closes the loop on interpersonal communication timing
Haptic assistance and warning design	[26,27,28]	Low-interference channels for urgent guidance and warning delivery	Mostly threshold-based or manually designed; not conditioned on neurocognitive readiness
MARL/CTDE coordination	[29,30,31]	Context-sensitive policy learning and coordinated multi-output control	No established dual-role neuro-adaptive formulation combining haptics and communication gating
Synchronization and neurodata governance	[32,33,34,35]	Timing precision for causal attribution; policy frameworks for neurodata handling	Rarely integrated into a single real-time adaptive system

**Table 2:** Synchronized modalities used in the proposed methodology.

Modality	Hardware/Source	Nominal Rate	Representation	Methodological Role
EEG	Emotiv EPOC X	256 Hz	Multichannel signal → spectral–topographic maps	Primary neurophysiological source for cognitive-state decoding and CLI estimation
Gaze/pupil	Eye-tracker (optional)	60 Hz	Gaze fixation and pupil descriptors $z_{t}^{gaze}$	Visual-attention context and optional multimodal augmentation
Telemetry	Simulator physics/events	100 Hz	Task-state descriptors $z_{t}^{tel}$	Hazard flags, task phase, kinematic context, progress indicators
Communication	Push-to-talk and message events	Asynchronous	Event history $z_{t}^{comm}$	Speech attempts, interruption timing, intervention history

**Table 3:** Main hyperparameters used for the CNN–LSTM decoder, the MAPPO controller, and the constrained optimization layer.

Component	Configuration
CNN spatial encoder	Conv2D(16, 3×3) + ReLU + MaxPool(2×2); Conv2D(32, 3×3) + ReLU + MaxPool(2×2); Conv2D(64, 3×3) + ReLU; spatial dropout =0.25
Temporal module	LSTM with 64 hidden units; temporal dropout =0.3
Temporal context	K=10 consecutive windows (≈1.9 s effective lookback)
Decoder outputs	Softmax head (CA/DA/SU) + sigmoid head (continuous CLI)
Decoder loss	Categorical cross-entropy + mean squared error (MSE)
Decoder optimizer	Adam, learning rate $=1\times 10^{-4}$, weight decay $=1\times 10^{-5}$
MAPPO discount factor	γ=0.99
GAE parameter	λ=0.95
PPO clipping ratio	ϵ=0.2
Entropy coefficient	0.01
Rollout horizon	2048 steps per environment
Mini-batch size	64
Optimization epochs/update	10
Stopping criterion	Reward variation below 5% for 100 consecutive iterations, or $5\times 10^{6}$ environment steps
Reward weights	$w_{s}=10.0,\;w_{p}=1.0,\;w_{c}=0.5,\;w_{l}=0.1,\;w_{h}=0.1$
Reward scales	α=1.0,β=5.0,η=0.1
Constraint budgets	$\epsilon_{load}=0.15,\;\epsilon_{cbe}=0.05$
Dual update rate	$\eta_{\lambda }=0.001$

**Table 4:** Distribution of labeled windows in the final chronologically partitioned dataset.

Class	Windows (n)	Percentage (%)	Reference Range (%)
Channelized Attention (CA)	325,000	65.0	60–70
Diverted Attention (DA)	125,000	25.0	20–25
Surprise/Startle (SU)	50,000	10.0	5–10
Total	500,000	100.0	—

**Table 5:** Decoder-level metrics for the CNN–LSTM model operating on spectral–topographic EEG maps, evaluated on the held-out chronological test partition.

Metric	Value
Overall accuracy	93.6%
Balanced accuracy	93.3%
Macro-F1	0.93
Recall (CA)	0.95
Recall (DA)	0.94
Recall (SU)	0.91

**Table 6:** Decoder ablation across input representations under the same chronological test protocol.

Input Representation	Accuracy	Balanced Acc.	Macro-F1	Interpretation
Raw channel-wise EEG windows	84.2%	83.8%	0.84	No explicit spatial prior; temporal modeling is preserved, but electrode geometry is implicit
Spectral feature vectors (band powers + FAA)	89.1%	88.7%	0.89	Compact and computationally efficient, but spatial organization is collapsed
Spectral–topographic EEG maps (ours)	93.6%	93.3%	0.93	Preserves spatial organization and temporal evolution; best aligned with the proposed representation-learning formulation

**Table 7:** Temporal-stability and runtime metrics for the online decoder and the full dual-loop pipeline.

Metric	Value
Adjacent-window class agreement	94.5%
Median $|{\hat{CLI}}_{t}-{\hat{CLI}}_{t-1}|$	0.025
End-to-end latency (window → actuation)	<50 ms
Minimum online throughput (full pipeline)	>20 windows/s
Real-time budget satisfied (Δ=100 ms)	Yes

**Table 8:** Inferential summary for the principal controller-level outcomes under the one-way repeated-measures ANOVA design. The 95% confidence intervals correspond to the mean reduction in the key Dual-loop vs. Open-loop paired contrast.

Outcome	ANOVA Result	Key Post Hoc Contrast	Effect Size	95% CI
Reaction time (RT)	F(3,72)=14.52, p<0.001	Dual-loop vs. Open-loop: p<0.001	$\eta_{p}^{2}=0.37$, d=1.12	[161.6,350.4]
CBE/session	F(3,72)=28.74, p<0.001	Dual-loop vs. Open-loop: p<0.001	$\eta_{p}^{2}=0.54$, d=1.85	[1.09,1.71]
CLI overload (%)	F(3,72)=8.31, p<0.001	Dual-loop vs. Open-loop: p=0.012	$\eta_{p}^{2}=0.25$, d=0.74	[2.92,10.28]
Engineer messages/session	F(3,72)=4.12, p=0.010	Dual-loop vs. Open-loop: p=0.045	$\eta_{p}^{2}=0.14$, d=0.45	[0.46,10.54]

**Table 9:** Joint operational outcomes across controller conditions (mean ± SD, N=25 pairs). CLI overload is the fraction of the session with ${\hat{CLI}}_{t}>\tau$.

Condition	RT (ms)	CBE/Session	CLI Overload (%)	Engineer Messages/Session
Open-loop (baseline)	487±156	4.1±1.3	14.2±4.3	18.4±5.2
Pilot-only	219±72	3.8±1.2	10.5±3.8	19.0±5.4
Engineer-only	482±150	3.1±1.1	13.8±4.1	14.2±4.8
Dual-loop	231±77	2.7±0.9	7.6±2.8	12.9±4.1

## Data Availability

The data presented in this study are available on request from the corresponding authors and subject to approval by the Research Ethics Committee of the University of Jaén due to the EEG and educational datasets generated and analysed during the current study contain information that could compromise the privacy of participants.

## Appendix A. Multi-Agent POMDP Formulation for Dual-Loop Control

To jointly optimize pilot assistance and engineer communication timing, the proposed dual-loop control framework is modeled as a partially observable Markov decision process with cooperative multi-output actions and centralized training with decentralized execution (CTDE). The environment is defined as

M=〈S,A,O,T,R,γ〉,

where the controller selects a joint action over two coordinated intervention channels: pilot-facing haptics and engineer-facing communication gating.

### Appendix A.1. Latent State Space (S)

The latent state $s_{t}\in S$ summarizes the coupled sociotechnical context:

(A1) $$s_{t}=\left[e_{t},\;z_{t}^{tel},\;z_{t}^{comm},\;z_{t}^{task}\right],$$

where $e_{t}$ denotes the pilot’s unobserved neurocognitive condition, $z_{t}^{tel}$ contains telemetry descriptors (e.g., hazard flags, segment type, kinematic context), $z_{t}^{comm}$ summarizes recent communication activity, and $z_{t}^{task}$ captures task-progression variables. The key point is that $e_{t}$ is not directly observed; instead, it must be inferred from synchronized neurophysiological measurements.

### Appendix A.2. Observation Space (O) and Neurostate Inference

The controller receives observations $o_{t}\in O$ constructed from multimodal synchronization across physiological, behavioral, and communication streams. Using the shared LSL time base, the synchronized windowed observation is written as

(A2) $$o_{t}=\left[x_{t:t+W}^{EEG},\;x_{t:t+W}^{tel},\;x_{t:t+W}^{comm},\;x_{t:t+W}^{gaze}\right],$$

where W=1 s is the window length and Δ=100 ms is the stride of the online loop.

Rather than feeding raw EEG windows directly into the policy, the framework first constructs spectral–topographic EEG maps. Let

(A3) $$M_{t}=\Phi \;\left(x_{t:t+W}^{EEG}\right)\in R^{32\times 32\times 4}$$

denote the map-construction operator that transforms each synchronized EEG window into a spatially organized spectral representation. The four map channels correspond to θ, α, β, and frontal alpha asymmetry (FAA). Over a temporal sequence of K=10 windows, the decoder receives

(A4) $$M_{t:t+K-1}=\left[M_{t},M_{t+1},\ldots ,M_{t+K-1}\right],$$

which provides an effective temporal context of approximately 1.9 s under the 1 s window/100 ms stride configuration.

A CNN–LSTM model then performs spatiotemporal neurostate inference:

(A5) $$\left({\hat{y}}_{t},{\hat{CLI}}_{t}\right)=f_{\theta }\;\left(M_{t:t+K-1}\right),\;{\hat{y}}_{t}\in \left\{\mathrm{CA},\mathrm{DA},\mathrm{SU}\right\},$$

where CA denotes *Channelized Attention*, DA denotes *Diverted Attention*, and SU denotes *Surprise/Startle*. The decoder may additionally output class posterior probabilities

$$p_{t}=P\left({\hat{y}}_{t}|M_{t:t+K-1}\right),$$

which can be used as optional confidence signals in implementation variants.

For policy input, we define the fused state vector

(A6) $$S_{t}=\psi \left(o_{t}\right)=\left[{\hat{CLI}}_{t},\;\mathrm{onehot}\left({\hat{y}}_{t}\right),\;z_{t}^{tel},\;z_{t}^{gaze},\;z_{t}^{comm}\right],$$

with $p_{t}$ treated as an optional auxiliary confidence descriptor when required by a specific implementation. This keeps the appendix aligned with the main-text formulation while preserving flexibility.

### Appendix A.3. Joint Action Space (A): Dual-Loop Control

The controller selects a joint action

(A7) $$a_{t}=\langle a_{t}^{pilot},\;a_{t}^{eng}\rangle$$

over two coordinated output channels:

(A8) $$\begin{array}{cc} a_{t}^{pilot} & \in A^{pilot}=\left\{\mathrm{OFF},\mathrm{LOW},\mathrm{HIGH}\right\}, \end{array}$$

(A9) $$\begin{array}{cc} a_{t}^{eng} & \in A^{eng}=\left\{\mathrm{OPEN},\mathrm{WARN},\mathrm{BLOCK}\right\}. \end{array}$$

The pilot action sets haptic guidance intensity, while the engineer action regulates communication timing through a traffic-light semaphore

$$g_{t}\in \left\{\mathrm{GREEN},\mathrm{YELLOW},\mathrm{RED}\right\}.$$

Operationally, WARN corresponds to a recommendation to delay or queue non-critical communication, whereas BLOCK corresponds to a strong “do-not-interrupt” signal. Human override is intentionally preserved for exceptional safety-critical cases.

To ensure conservative behaviour during acute neurocognitive perturbations, the learned gate is combined with a minimal safety override:

(A10) $$g_{t}\leftarrow \left\{\begin{array}{cc} \mathrm{RED}, & {\hat{y}}_{t}=\mathrm{SU}\;\mathrm{or}\;{\hat{CLI}}_{t}\geq \kappa_{red},\\ g_{t}, & \mathrm{otherwise}. \end{array}\right.$$

### Appendix A.4. Reward and Safety-First, Constrained Optimization

The instantaneous reward follows the formulation in Equation (13) of the main text:

(A11) $$r_{t}=w_{s}\;r_{t}^{safety}+w_{p}\;r_{t}^{progress}+w_{c}\;r_{t}^{comm}-w_{l}\;{\hat{CLI}}_{t}-w_{h}\;I\left[a_{t}^{pilot}=\mathrm{HIGH}\right],$$

with the coefficients instantiated as

$$w_{s}=10.0,\;w_{p}=1.0,\;w_{c}=0.5,\;w_{l}=0.1,\;w_{h}=0.1,$$

and

α=1.0,β=5.0,η=0.1.

Accordingly, the communication term is defined around semaphore compliance:

(A12) $$r_{t}^{comm}=+I\left[u_{t}=1\wedge g_{t}=\mathrm{GREEN}\right]-\beta \;I\left[u_{t}=1\wedge g_{t}=\mathrm{RED}\right]-\eta \;I\left[u_{t}=0\wedge g_{t}=\mathrm{GREEN}\right],$$

where $u_{t}$ indicates an engineer message attempt (push-to-talk onset) at time *t*.

To enforce *safety-first* behavior, overload exposure and mistimed communication events are additionally constrained, as defined in Equation (20). Using an overload threshold τ on the continuous CLI, the cost indicators are

(A13) $$d_{t}^{load}=I\left[{\hat{CLI}}_{t}>\tau \right],\;d_{t}^{cbe}=I\left[u_{t}=1\wedge g_{t}=\mathrm{RED}\right],$$

with corresponding episodic costs

(A14) $$C^{load}\left(\pi \right)=E_{\pi }\left[\frac{1}{T}\sum_{t=0}^{T-1}d_{t}^{load}\right],\;C^{cbe}\left(\pi \right)=E_{\pi }\left[\sum_{t=0}^{T-1}d_{t}^{cbe}\right].$$

In the present implementation, the constraint budgets were set to

$$\epsilon_{load}=0.15,\;\epsilon_{cbe}=0.05,$$

and the projected dual update used

$$\eta_{\lambda }=0.001.$$

Optimization is performed using the primal–dual Lagrangian in Equation (21) together with the projected multiplier update of Equation (22).

Interpretive note.

Overload is *not* modeled as an additional discrete class. Instead, it is defined operationally through the continuous condition

$${\hat{CLI}}_{t}>\tau ,$$

which directly drives RED gating, constrained costs, and safety-aware control.

### Appendix A.5. Transition Dynamics (T) and Sociotechnical Coupling

Transition dynamics

$$T(s_{t+1}|s_{t},a_{t})$$

describe how interventions alter both pilot neurocognitive evolution and task trajectory. Under elevated CLI or acute *Surprise/Startle*, reducing non-critical communication can decrease the probability of further attentional destabilization, while timely haptics can shorten reaction time and reduce near-miss risk under visual saturation. This coupling is what motivates the dual-loop control formulation: an intervention that benefits one role (e.g., engineer silence) is only useful insofar as it preserves pilot stability, responsiveness, and safety margins.

Note on interpretation. The POMDP formalism models the AI as a mediator of timing and channel contention. It is not intended to replace human judgment; rather, it provides real-time coordination signals under strict purpose limitation for safety and communication timing.

## Appendix B. Cognitive Load Index (CLI) Derivation

This appendix details the computation of the Cognitive Load Index (CLI) used throughout the paper as a continuous proxy for workload and cognitive saturation. The CLI is designed to be (i) computable in real time from portable EEG, (ii) robust to inter-individual variability through participant-specific normalization, and (iii) compatible with both the spectral–topographic EEG representation and the downstream dual-loop control policy.

### Appendix B.1. Preprocessing and Windowing

EEG is segmented into sliding windows of length W=1 s with stride Δ=100 ms. Each window undergoes artifact attenuation through Artifact Subspace Reconstruction (ASR), using the warm-up phase as the clean reference covariance estimate. The signal is then processed with a zero-phase FIR band-pass filter between 1 Hz and 40 Hz and a zero-phase FIR notch filter centered at 50 Hz with a 49–51 Hz stopband. Finally, each channel is standardized prior to spectral estimation. These steps are executed within the same multimodal synchronization loop used by the main pipeline, so the resulting CLI remains temporally aligned with telemetry, gaze, and communication events.

### Appendix B.2. Spectral Biomarkers

For each channel $c\in \{1,\ldots ,N_{c}\}$, power spectral density is estimated using Welch’s method with a Hann window, segment length $n_{\mathrm{perseg}}=128$ samples (0.5 s), and 50% overlap ($n_{\mathrm{overlap}}=64$ samples). Band powers are then integrated as

(A15) $$P_{b}\left(c,t\right)=\int_{f\in b}PSD_{c}\left(f,t\right)\;df,\;b\in \left\{\theta ,\alpha ,\beta \right\},$$

with frequency bands

θ:4–8Hz,α:8–12Hz,β:13–30Hz.

This parameterization yields a frequency resolution of 2 Hz, which is appropriate for robust extraction of the broad workload-related bands used in the study.

Band powers are then aggregated across channels using robust averaging:

(A16) $${\bar{P}}_{b}\left(t\right)=\mathrm{robust}\_{\mathrm{mean}}_{c}\left(P_{b}\left(c,t\right)\right).$$

### Appendix B.3. Frontal Alpha Asymmetry (FAA)

To characterize diversion- and withdrawal-related dynamics, frontal alpha asymmetry is computed from electrodes F3 and F4:

(A17) $${\mathrm{FAA}}_{t}=\ln\;\left(P_{\alpha }\left(F4,t\right)\right)-\ln\;\left(P_{\alpha }\left(F3,t\right)\right).$$

FAA is used both as an auxiliary feature for CLI estimation and as the fourth channel in the spectral–topographic representation.

### Appendix B.4. Raw CLI Construction

A raw workload score is defined as a weighted combination of standardized biomarkers:

(A18) $$CLI_{t}^{raw}=\lambda_{1}\;z\;\left({\bar{P}}_{\theta }\left(t\right)\right)-\lambda_{2}\;z\;\left({\bar{P}}_{\alpha }\left(t\right)\right)+\lambda_{3}\;z\;\left({\bar{P}}_{\beta }\left(t\right)\right)+\lambda_{4}\;z\;\left({\mathrm{FAA}}_{t}\right),$$

where z(·) denotes per-participant standardization estimated from warm-up segments, and $\lambda_{i}$ are calibration weights fixed after warm-up.

This formulation reflects the usual interpretation that increased frontal theta and beta may indicate elevated task engagement or strain, whereas reduced alpha is typically associated with increased attentional demand. FAA contributes an asymmetry-sensitive term that helps distinguish diversion-related dynamics from more globally channelized or startle-related states.

### Appendix B.5. Robust Normalization to the Unit Interval

To reduce sensitivity to outliers and between-subject baseline shifts, robust normalization is applied:

(A19) $${\tilde{CLI}}_{t}=\mathrm{clip}\left(\frac{CLI_{t}^{raw}-\mathrm{median}\left(CLI^{raw}\right)}{\mathrm{IQR}(CLI^{raw})},-3,3\right),$$

where IQR denotes the interquartile range. The clipped value is then mapped to the unit interval:

(A20) $${\hat{CLI}}_{t}=\frac{{\tilde{CLI}}_{t}+3}{6}.$$

The resulting normalized index

$${\hat{CLI}}_{t}\in \left[0,1\right]$$

is the quantity used throughout the main text for thresholding, overload estimation, and policy input.

### Appendix B.6. Relation Between CLI and Spectral–Topographic EEG Representations

The CLI is derived from the same synchronized EEG window used to construct the spectral–topographic EEG maps. Conceptually, both outputs describe the same underlying neurophysiological segment but at different levels of abstraction. The topographic maps preserve the spatial distribution of spectral activity and are provided to the CNN–LSTM decoder, whereas the CLI acts as a compact continuous summary of workload intensity. This dual representation is useful for dual-loop control: the map sequence supports rich class inference, while the CLI provides a stable scalar control variable for thresholding, cost construction, and overload exposure analysis.

### Appendix B.7. Thresholding for Gating and Policy Input

During the 5 min warm-up phase, the continuous CLI was recorded while each participant performed the baseline task. For each subject, the intra-subject mean μ and standard deviation σ of the CLI were computed. The moderate overload threshold was defined as

(A21) $$\kappa_{yel}=\tau =\mu +1.5\sigma ,$$

whereas the critical threshold for communication blocking was set to

(A22) $$\kappa_{red}=\mu +2.0\sigma .$$

These thresholds define moderate and extreme load regions consistent with the semaphore logic of the main text. The final policy input vector is therefore written as

(A23) $$S_{t}=\mathrm{Norm}\left([{\hat{CLI}}_{t},\;\mathrm{onehot}\left({\hat{y}}_{t}\right),\;z_{t}^{tel},\;z_{t}^{gaze},\;z_{t}^{comm}]\right),$$

where Norm(·) denotes the final scaling step used before policy inference.

Interpretive note. The CLI should not be understood as a direct measure of “true” cognitive load in a psychological absolute sense. Rather, it is an operationally calibrated continuous index that supports real-time gating, overload exposure analysis, and safety-first control within the proposed framework.
